# Structural basis for Mg²⁺–glycine synergy in the tandem glycine riboswitch revealed by cryo-EM

**DOI:** 10.1038/s44511-026-00002-y

**Published:** 2026-10-01

**Authors:** Nathan Jespersen, Jigneshkumar Dahyabhai Prajapati, Ankush Singhal, Karissa Y. Sanbonmatsu

**Affiliations:** 1 Theoretical Biology and Biophysics, Theoretical Division, Los Alamos National Laboratory, Los Alamos, NM USA; 2 New Mexico Consortium, Los Alamos, NM USA

**Keywords:** Biochemistry, Biophysics, Structural biology

## Abstract

Riboswitches are non-coding RNA sequences that control cellular processes through ligand binding. Conformational heterogeneity is fundamental to riboswitch functionality, yet this same attribute makes structural characterization of these mRNA elements challenging. Here, we use cryo-electron microscopy to characterize different structural populations of the glycine riboswitch tandem aptamers, and molecular dynamics simulations to support the observed ligand orientation in the holo state. We find that Mg^2+^ enables formation of the fully folded “walking man” conformation, which consists of a rigidified core and two dynamic helices, but is insufficient on its own to make this the dominant state. In Mg²⁺ conditions alone, approximately one-third of the picked particles are fully folded, whereas two thirds adopt distinct, partially folded states. Glycine interactions further stabilize the fully folded conformation, doubling the relative population of fully folded particles by stabilizing a conserved inter-aptamer Hoogsteen base pair, enabling our capture of a 2.9 Å-resolution structure for this RNA-only system. The population data show that glycine and Mg²⁺ operate synergistically: glycine enhances Mg²⁺ localization, while Mg^2+^ drives glycine recruitment. Our findings indicate that cryo-electron microscopy offers a promising avenue to characterize RNA folding ensembles.

## Introduction

RNAs are structurally and functionally heterogeneous molecules that are utilized by all known organisms. In bacteria, RNA accounts for nearly 20% of the entire dry mass of the cell^1^. Despite their abundance, RNA structure is still poorly characterized and difficult to predict when compared to protein structure^2^. Indeed, of the >241,000 structures in the PDB in August of 2025, less than 1% are RNA-only structures, and a small fraction of those represent complex structures under 3 Å resolution^3^. High-resolution RNA structure determination is hindered by local and global heterogeneity, which complicates single-particle averaging and limits attainable resolution. RNA secondary and tertiary structures are also surprisingly malleable and prone to kinetic trapping in local free energy minima^4^. Their folds are often highly dependent on solution conditions^5–8^ or minor differences in transcriptional start sites^9^. Consequently, RNA structural characterization methods frequently produce either complex data resulting from the convolved signals of divergent states (as in the case of 2D probing methods or NMR), or a single snapshot of a dynamic system (as in the case of X-ray crystallography). While these methods are extraordinarily useful for describing some aspects of RNA structure, recent advancements in cryo-electron microscopy (cryo-EM) data collection and processing have made it possible to simultaneously characterize heterogeneity and produce high-resolution information^10–13^, while removing signal from misfolded particles.

Riboswitches are RNA-based regulatory elements residing in mRNA that undergo structural changes in response to specific metabolites like glycine^14^, guanine^15^, SAM^16^, and many others. Structural heterogeneity is therefore fundamental to riboswitch functionality. Riboswitches are typically located in 5′-untranslated regions of mRNA, where they operate in *cis* to either transcriptionally or translationally regulate the expression of downstream genes^17,18^. Notably, riboswitches often control genes that metabolize or transport the very compounds that bind to them, creating an extremely efficient feedback loop. Riboswitches generally consist of a ligand-binding aptamer domain, and a regulatory region, called the expression platform. Aptamer domain interactions with the ligand trigger global rearrangements in riboswitch structure, leading to either upregulation (ON-switch) or downregulation (OFF-switch) of target genes^18^. In some cases, such as in the glycine riboswitch family, homologous ON and OFF switches have both been identified, with downstream genes being evolutionarily linked to the regulatory mechanism^19^.

**Fig. 1:**
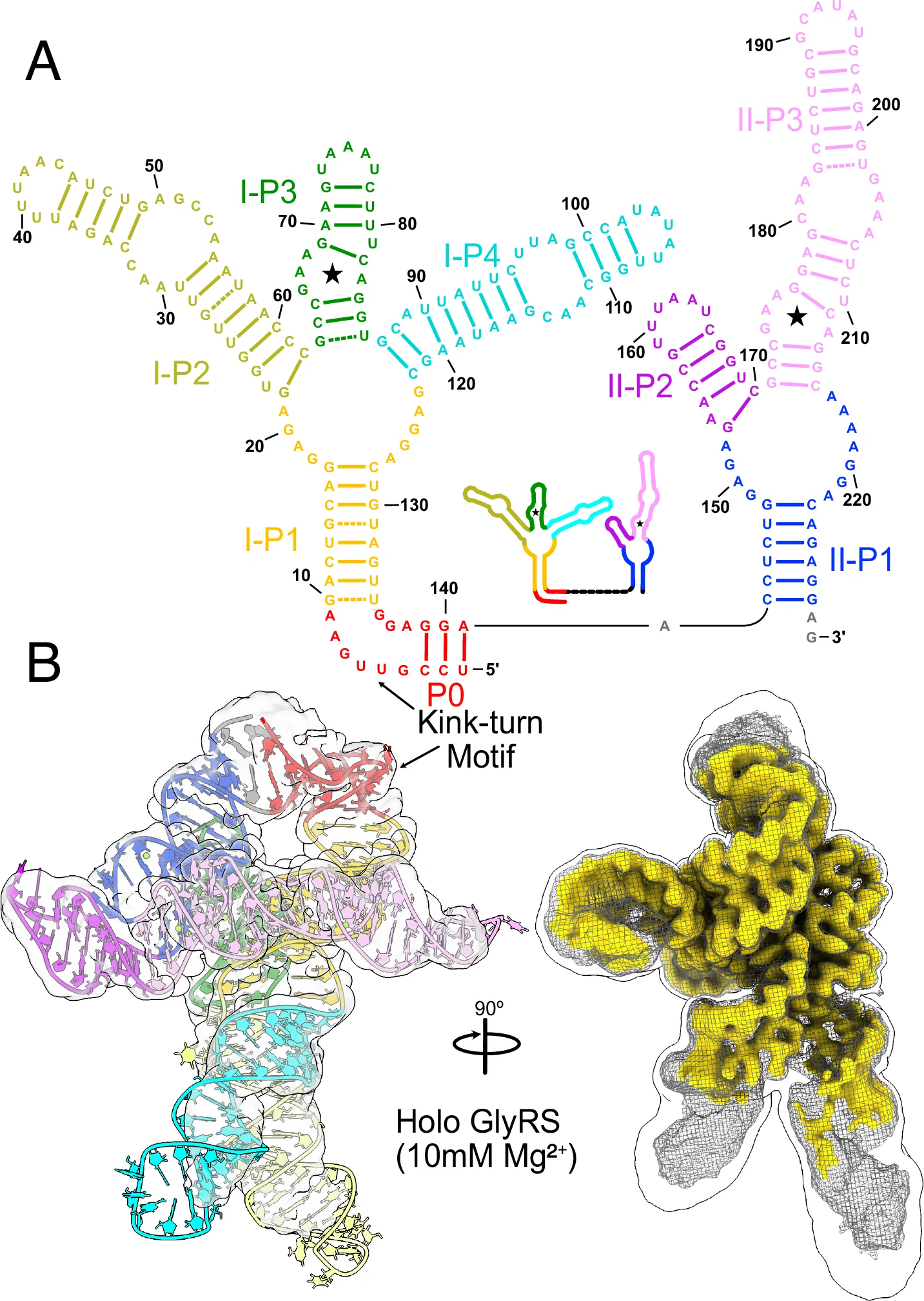
Overall walking man fold for the holo glycine riboswitch. **A** Secondary structural representation of the tandem glycine riboswitch aptamers, colored to highlight structural elements. Stars denote glycine binding pockets. A schematic representation is included as an inset. **B** Cryo-EM density of the holo glycine riboswitch structure, with the model colored consistent with (**A**). A triple overlay of raw (mesh), sharpened (gold), and Gaussian-filtered (silhouette) maps is shown on the right to demonstrate how different regions of the model were fit and refined.

Glycine riboswitches exhibit unusual functional and mechanistic diversity, extending beyond their simple ON/OFF role. Different homologs can function as either transcriptional or translational riboswitches and may contain either a single glycine-binding aptamer domain or a pair of aptamer domains (Fig. 1A). Riboswitches with two aptamer domains are referred to as “tandem riboswitches” and their functional states are often described using Boolean logic^20^. In the case of the glycine riboswitch, where both aptamers bind glycine molecules to regulate a shared expression platform, an AND logic gate is typically ascribed. This would mean that each aptamer must bind a glycine molecule to effectively regulate the downstream gene.

The distinctive architecture of the tandem glycine riboswitch has long intrigued riboswitch researchers, leading to extensive biochemical and biophysical investigations^14,19,21–27^. In particular, the *Vibrio cholerae* glycine riboswitch has been the subject of detailed thermodynamic and structural work, including SAXS and hydroxyl-radical footprinting experiments that revealed ligand- and Mg²⁺-dependent compaction of the tandem aptamer domains^25^. Site-directed mutagenesis and nucleotide-analog interference mapping (NAIM) in this system have identified key residues in P1 and P3 helices that modulate glycine affinity^22^. Notably, phosphorothioate substitutions at backbone positions implicated in metal coordination reduced glycine affinity, consistent with a functional role for specific Mg²⁺ interactions within the binding pockets and at inter-aptamer contacts. Initial investigations also demonstrated that some glycine riboswitch (glyRS) constructs displayed cooperative glycine-binding behavior, where binding of glycine to one aptamer increased the affinity of the other aptamer for glycine^14,22,23^. However, subsequent research identified a conserved kink-turn interaction between a leader and linker region, which inhibits glycine binding cooperativity and facilitates both riboswitch folding and glycine interactions^28,29^. These findings led to the suggestion that the observed glycine binding cooperativity might have been due to the truncated constructs. The prevailing model posits that the function of the tandem glycine riboswitch involves a complex interplay between aptamer dimerization and glycine binding. Detailed analyses via equilibrium dialysis assays and site-directed mutagenesis indicate that a single aptamer is sufficient to drive glycine binding, and this interaction in turn stabilizes the tertiary structure of the riboswitch, facilitating gene regulation^19,21,26,27^. Importantly, RNA folds for riboswitches are also highly sensitive to the electrostatic environment of the solution due to their intricate tertiary folds and complex interactions with Mg^2+^ ions^30,31^. Cooperativity in riboswitches may be attributed to Mg^2+^ interactions leading to increased affinity for target ligands^8,32^. Understanding how Mg^2+^ and other cations impact the populations of various conformational states of the ensemble is therefore imperative to understand RNA folding.

Here, we use a combination of cryo-EM and molecular dynamics simulations (MD) to characterize and depict the structural diversity inherent in the tandem aptamer domains of the *V. cholerae* glycine riboswitch, a tandem OFF riboswitch, in three conditions: (i) without ligand or divalent cation, (ii) in the presence of Mg^2+^, and (iii) with both Mg^2+^ and glycine. Our analyses led to a “walking man” structure for the fully folded glycine riboswitch (Fig. 1B), resolvable to 2.9 Å in the holo complex and to 3.3 Å in the absence of glycine. By picking particles without stringent size restrictions, we were able to generate a comparative ensemble of maps for each condition. This method revealed several distinct folds that are stable in the presence of Mg^2+^ and partially stabilized in the absence of Mg^2+^. While Mg^2+^ appears to shift the equilibrium toward folded conformations, glycine shifts the population of fully folded glycine riboswitch from approximately one to two thirds. Indeed, by comparing the differences in the electrostatic potential maps in the presence or absence of glycine, we show that a conserved inter-aptamer Hoogsteen base pair is held in a more consistent and proximal arrangement in the presence of glycine, leading to stabilization of the entire riboswitch fold.

Within the active sites, binding orientations for the glycines were fit based on the cryo-EM density and key interactions were supported by explicit solvent MD, providing a replicable method for combining mid-level resolution with MD to obtain biophysically reliable structures. Both glycine binding sites contain notable density for a coordinated Mg^2+^ that directly interacts with the carboxyl groups of the glycines. This Mg^2+^ density is either unidentifiable, or greatly reduced in the absence of glycine, supporting previous suggestions that there is heterotropic cooperative binding between Mg^2+^ and glycine^32^. Our data provide atomic-level details about the structure and dynamics of the glycine riboswitch, and demonstrate how key ligands modulate the stability of various populations within an RNA’s structural ensemble.

## Results

### Holo state of the glycine riboswitch adopts “walking man” fold

To better categorize populations within the folding ensemble, it is important to have a well-resolved reference structure to align partially folded arrangements. Previous small-angle X-ray scattering studies by Lipfert et al. (2007, 2010) have produced low-resolution descriptions of the tandem glycine riboswitch, which demonstrated that it is most stable and compacted in the presence of both Mg^2+^ and glycine^25,32^. We therefore first sought to characterize the *V. cholerae* glycine riboswitch in a solution containing 10 mM Mg^2+^ and 2 mM glycine to obtain a reasonably homogeneous sample. Under these conditions, we were able to generate a 2.9 Å electrostatic potential map for the holo glycine riboswitch, which folds into a conformation resembling a “walking man” (Fig. 1B and SI Fig. 1). The core region of this fold (Aptamer-I P1 and P3; Aptamer-II P1 and P3) is well resolved, with clear density around base pairs. In contrast, the arm (Aptamer-II P2 and P3) and leg regions (Aptamer-I P2 and P4) are not as well resolved (Fig. 1B and Supplementary Fig. 3). This may be due to the relatively small size and multidirectional stepwise motion of the leg regions (described below).

**Fig. 2:**
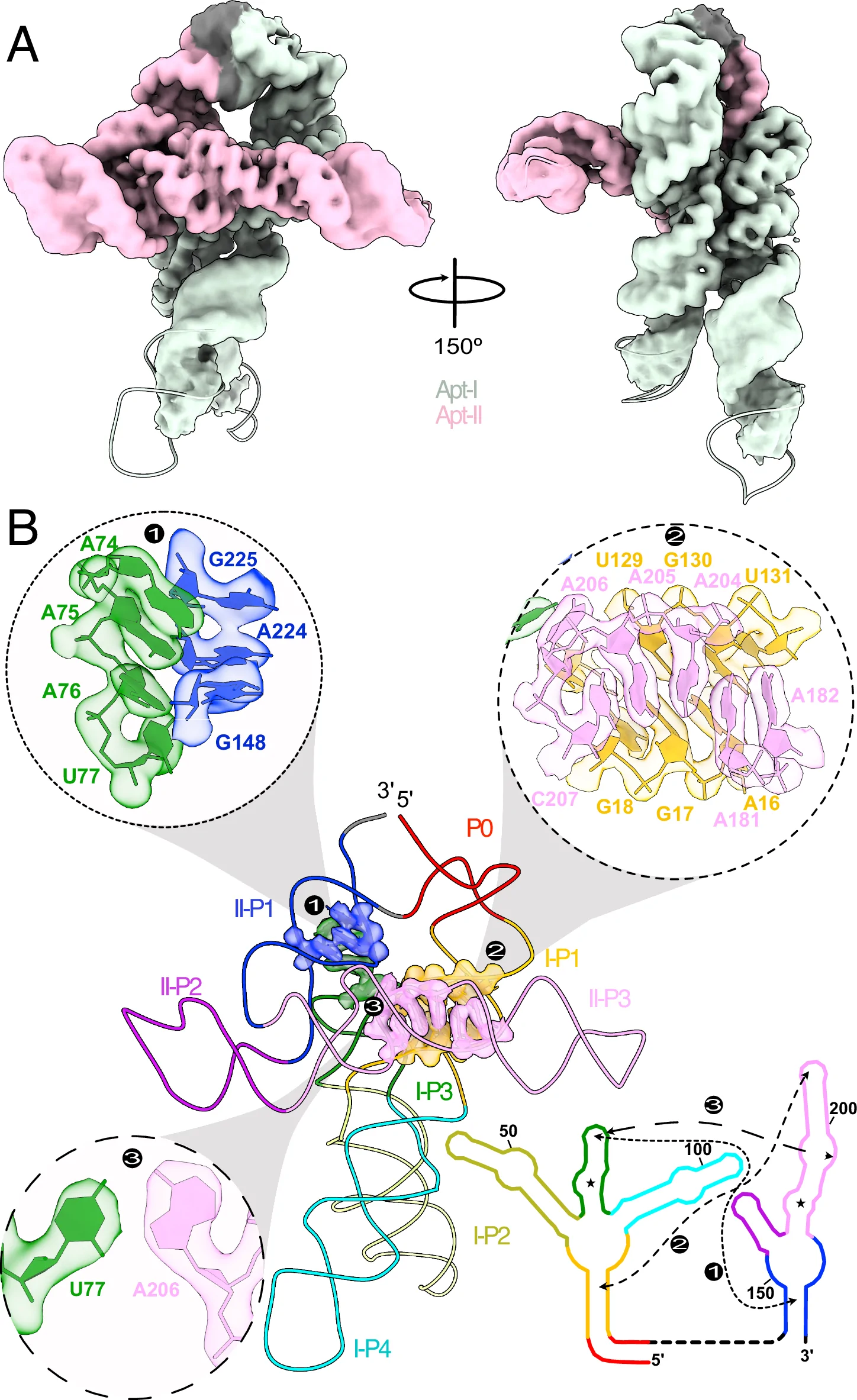
The full glycine riboswitch fold is stabilized by three main regions of inter-aptamer contacts. **A** Cryo-EM map and model colored to highlight residues derived from Apt-I (light green), Apt-II (pink), or linker regions (gray). **B** Regions of Inter-aptamer contact. Residues of one aptamer that approach within 2.5 Å of the other aptamer were considered as a contact, leading to three regions involved in stabilization of the fully folded conformation. This includes two sets of A-minor motifs (*1,2*), and one Hoogsteen base pair (*3*). Cryo-EM density for contact regions is extracted to demonstrate fit. A schematic representation of these contact regions is included (right) to emphasize regions involved in stabilization of the full fold. Model and schematic representation are colored to emphasize structural elements.

Although it can be difficult to build initial models for RNA-only systems without collecting 2D probing data^33,34^, prior work on a holo system for a different species (*F. nucleatum*), which did not explore the conformational ensemble, yielded a 3.55 Å crystal structure of the glycine riboswitch stabilized by a proteinaceous partner. An excellent previous study also used cryo-EM to obtain a 5.7 Å structure of the holo *V. cholerae* glycine riboswitch, which is 35% longer than the *F. nucleatum* homolog^23,33^. While 5.7 Å is insufficient resolution to make out finer molecular details, this structure, in combination with the *F. nucleatum* X-ray crystallography structure, served as excellent starting points to build our model (Fig. 1B).Our cryo-EM study shows that the tandem aptamers of the *V. cholerae* glycine riboswitch adopt distinct folds, with aptamer one (Apt-I) forming a 4-way junction to fashion the back and leg helices, while aptamer two (Apt-II) forms a three-way junction to create the arms and head region (Figs. 1 and 2). The global fold is stabilized by three main inter-aptamer interaction zones: (1) three adenines in the loop of Apt-I P3 buried in the minor groove of Apt-II P1, (2) the reciprocal interaction with four adenine bases of Apt-II P3 binding to a loop in Apt-I P1, and (3) a conserved Hoogsteen base pair (U77-A206 here) between the P3 regions^23^ (Fig. 2). Our findings are consistent with previous chemical probing and crystallography work, which identified these “A-minor” motifs (Supplementary Fig. 3D) and demonstrated that P1 and P3 are conserved, while P2 and P4 are variable^21–24,27^. The kink-turn motif is positioned to contribute to tandem aptamer stabilization. We visualize this region and confirm that nucleotides G4-A8 adopt a conformation consistent with a kink-turn motif (RNA Suite code: **7r6p2[0a**^35^; Supplementary Fig. 4). Interestingly, the regions of the *V. cholerae* glycine riboswitch that are best resolved (Fig. 1B and Supplementary Fig. 3) mirror those that are structurally most conserved among the *V. cholerae* and *F. nucleatum* glycine riboswitches (Supplementary Fig. 5). These regions also display the highest level of sequence conservation across all known homologs (Supplementary Fig. 5)^21^.

### Exploring the conformational ensemble by investigating non-holo states: glycine remodels the conformational landscape, stabilizing the fully folded conformation

**Fig. 3:**
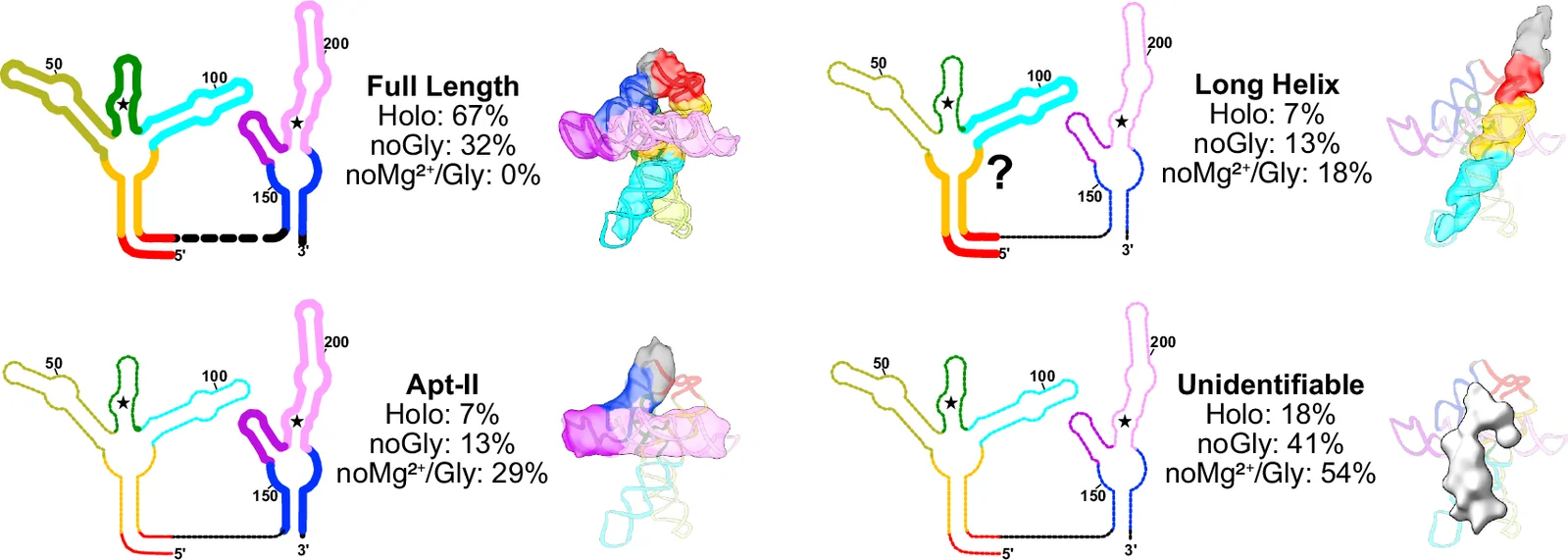
Populations of glycine riboswitch folds are highly responsive to solution conditions. Four general classes of structures are shown as both a schematic representation and with a representative transparent map overlaid with a potential structural element from the model. Fractional populations within the holo, no glycine, and no glycine/Mg^2+^ samples are noted for each class. A question mark is used to emphasize the uncertainty inherent in defining which specific residues are involved in the structure. The percentage in each circumstance is derived from populations shown in Supplemental Fig. 1. Similar classes were grouped when they displayed high correlation.

To understand how glycine and Mg^2+^ affect the ensemble of configurational populations of the glycine riboswitch, we picked particles from cryo-EM grids in three conditions: (1) holo glycine riboswitch with 10 mM Mg^2+^ and 2 mM glycine, (2) glycine riboswitch with 10 mM Mg^2+^ without glycine, and (3) glycine riboswitch without Mg^2+^ and without glycine. Particles were used to generate ten ab initio models for each condition, which were then heterogeneously refined into ten representative structures of the conformational ensemble, using cryoSPARC’s heterogeneous refinement job^36^. When glycine and magnesium are both present, approximately two thirds of all particles are fully folded (Fig. 3 and Supplementary Fig. 1). In addition, one fold reminiscent of Apt-II alone is identifiable (Fig. 3 and Supplementary Fig. 1). Our results are consistent with those of previous studies that show that Apt-II is the driver for glycine binding in *V. cholerae* and the structurally more stable aptamer^21,24,27^.

The population of fully folded RNA is significantly lower when glycine is absent (Fig. 3 and Supplementary Fig. 1), accounting for only one-third of all picked particles. Instead, the relative population of the potential Apt-II fold is doubled (Fig. 3 and Supplementary Fig. 1). The strong enrichment of fully folded particles when both ligands are present suggests that glycine helps to stabilize inter-aptamer contacts to shift the single aptamer populations towards the fully folded state.

Nearly 41% of particles belong to unidentifiable classes in the Mg^2+^-alone sample, compared to 18% in the holo sample (Fig. 3 and Supplementary Fig. 1). While these low-resolution structures could be artifacts evolving from insufficient statistical coverage, the shift of populations upon addition of ligands suggests that they are instead intermediates or off-target, misfolded conformations. In some cases, similar but unidentifiable folds are present in both samples containing Mg^2+^ (Supplementary Fig. 1). It may therefore be possible to use cryo-EM to characterize potentially relevant conformations in RNA folding pathways. We note, however, that these in vitro samples do not reproduce the crowded intracellular environment, the full complexity of physiological ion conditions, or co-transcriptional folding by the polymerase. All of these factors are likely to be of great importance for describing physiologically meaningful folding pathways.

### Synergy between Mg^2+^ and glycine: the glycine riboswitch requires Mg^2+^ to adopt the fully folded conformation

In contrast to the cases discussed above, no fully folded particles were discernable in the absence of both Mg^2+^ and glycine (Supplementary Fig. 1 and Fig. 3). Nearly 30% of all picked particles instead adopted a “seven-shaped” fold, akin to a portion of the Apt-II structure (Fig. 3 and Supplementary Fig. 1). The relatively lower resolution of maps from these classes indicates that the RNA is dynamic, leading to poor signal averaging. Interestingly, long RNA helices were identifiable in all three conditions, with the population decreasing as the fully folded population increased (Fig. 3). These data suggest that either a single long helix, such as the one encompassing P1 and P2 of Apt-I, is particularly stable, or that RNA chains form helical assemblies in solution to facilitate burial of bases.

### The glycine riboswitch is highly heterogeneous in the leg regions when fully folded

**Fig. 4:**
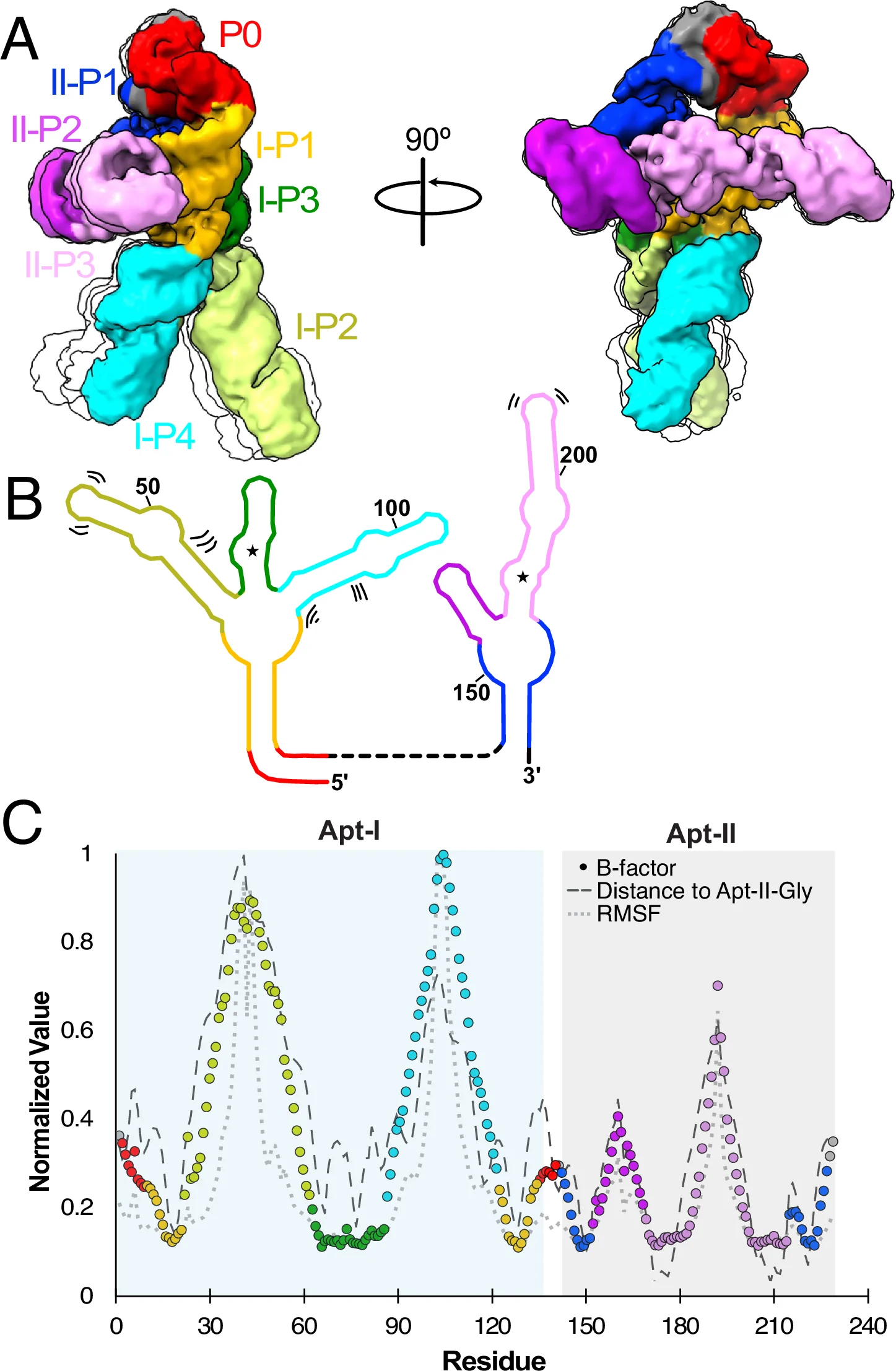
The glycine riboswitch P2 and P4 regions are highly dynamic. **A** Four representative models derived from 3D classification of the final holo particle pool. One structure is colored based on structural elements, while three others are shown as simple transparent silhouettes. **B** Schematic representation of secondary structure, with bending and shifting motions denoted by sets of two or three lines based on the intensity and directionality of the motion. **C** Normalized *B*-factor (colored circles), normalized distance of Apt-II glycine (dashed line, dark gray), and RMSF from MD simulations (dashed line, light gray) per residue for the holo glycine riboswitch cryo-EM structure. Circles are colored consistent with structural elements in (**A**, **B**). Apt-I and Apt-II regions are shaded in blue and gray, respectively.

The degrees of freedom in the RNA backbone manifest not only an ensemble of folds, but also dynamic regions within the folds. To characterize glycine riboswitch dynamics and identify potential hinge regions, we used two approaches: (i) a 3D classification and 3D-variability analysis on our final holo particle pool (Supplementary Movies 1 and 2 and Fig. 4)^37^ and (ii) structure-based molecular simulations. With the first method, we identified three distinct movements within the *V. cholerae* glycine riboswitch. First, we observe that the distance between the feet regions increases from 51 to 61 Å, and their tangent-tangent angle increases from 51^o^ to 81^o^. The overall motion of the leg regions resembles a stepping motion (Supplementary Movie 1). Second, the core hip regions flex simultaneously, leading to the legs pivoting forward by ~9^o^ (Supplementary Movie 2). The final motion visible is within the head region, where our low-resolution analysis catches movement in the 3’ and 5’ ends of the RNA (Supplementary Movies 1 and 2). These movements are partially recapitulated in the PHENIX-derived B-factors, which show that the legs are the most dynamic region (Fig. 4). Interestingly, the normalized B-factors correlate well with the distance between each nucleotide and the Apt-II glycine. The glycine binding site is therefore likely to be the most stable part of the holo riboswitch. Although it is unclear what role the more dynamic regions play in riboswitch functionality, it is of note that the most dynamic portion (i.e., Apt-I P4) is present in only 13% of glycine riboswitches^21^.

To further evaluate the dynamic motions obtained via cryo-EM, we used all-atom structure-based molecular simulations. The RMSF values mirrored the PHENIX-derived B-factors (Fig. 4C), and the two distinct stepping motions seen in cryo-EM were mirrored in the principal component analysis motions derived from the molecular simulations (Supplementary Movies 3 and 4 and Supplementary Fig. 6), indicating that molecular simulations can capture molecular motions similar to those derived from cryo-EM variability analysis.

### Glycine and Mg^2+^ bind in a synergistic manner and stabilize inter-aptamer contacts

**Fig. 5:**
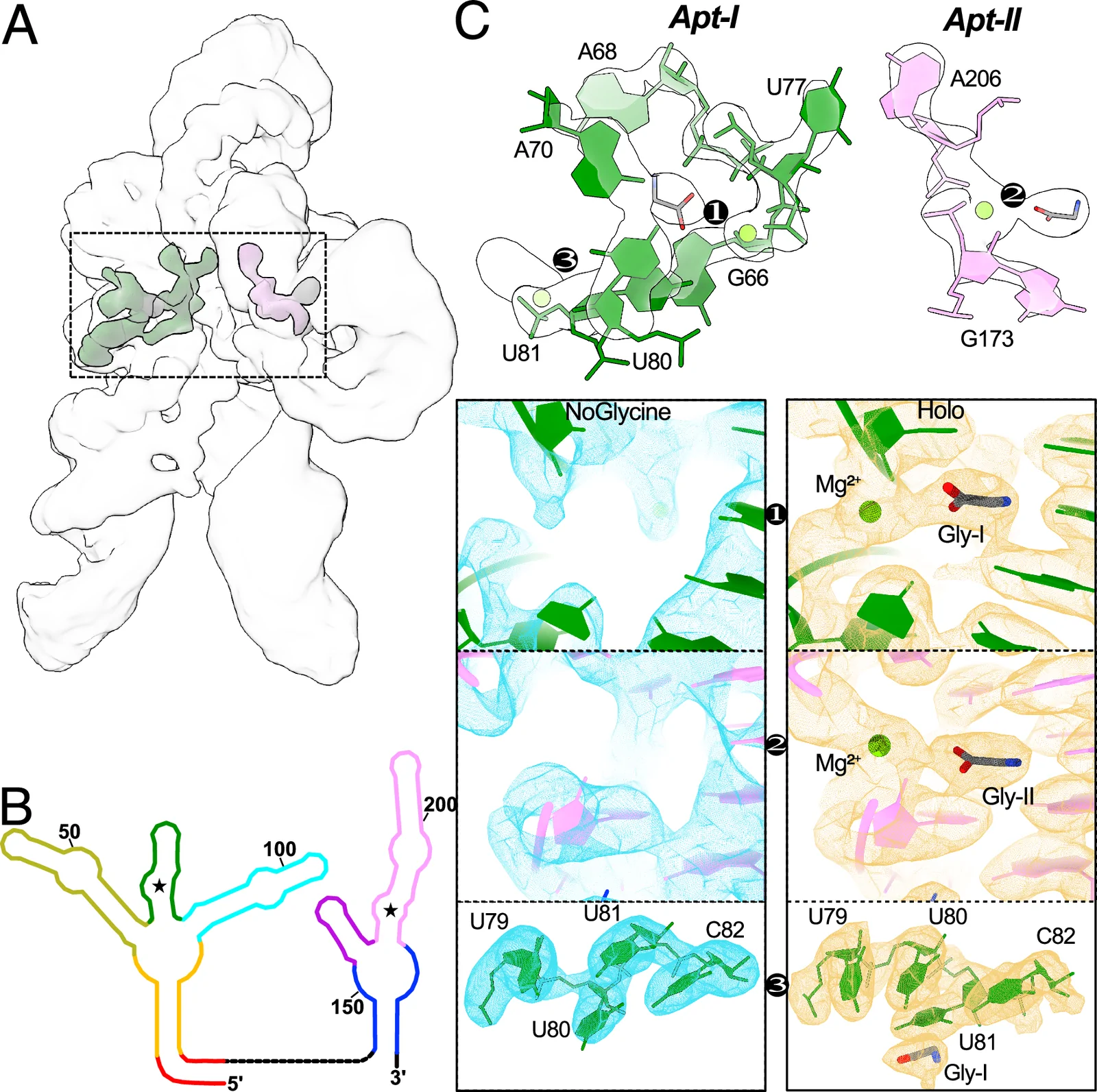
Glycine and magnesium synergistically stabilize inter-aptamer glycine riboswitch contacts. **A** Transparent cryo-EM map of the holo glycine riboswitch, overlaid with a Gaussian-filtered difference map of the holo glycine riboswitch minus the apo glycine riboswitch, demonstrating structural changes are localized to the glycine binding loci. **B** Schematic representation of the glycine riboswitch secondary structure, colored to emphasize different structural elements. **C** Enlarged view of regions altered in the presence of glycine, with residues colored consistent with panel (**B**) and overlaid with a silhouette view of the difference map. Glycine ligands are colored in gray and magnesium ions are shown in lime green. Note the direct path of stabilization between the two glycine residues, connected via the conserved inter-aptamer Hoogsteen base pair (here, U77-A206). Three key regions are further enlarged, with modified density in the Apt-I (*1*), and Apt-II (*2*) binding pockets, demonstrating a cooperative interaction between magnesium and glycine in these regions. Movement of U80 and U81 (*3*) may capture the movement required to allow glycine access to the tight-binding pocket.

The conformational ensemble analysis demonstrated that glycine stabilizes the fully folded conformation. To understand the mechanism of stabilization, we solved the glycine riboswitch structure for the most enriched class in the presence of 10 mM Mg^2+^ and compared it to the holo structure. Notably, despite using the same RNA preparation and a similar number of particles (216 K in the holo sample and 207 K with Mg^2+^ alone), the resolution was significantly lower in the absence of glycine, yielding a 3.3 Å structure. The overall fold for our RNA construct, which lacks the regulatory expression platform, is essentially identical under the two conditions (Supplementary Fig. 1). Indeed, a difference map of the electrostatic potentials under either condition demonstrates that all the major changes take place in a localized region surrounding the glycine binding sites (Fig. 5).

As depicted in the RNA secondary structure diagram (Fig. 1A), the two glycine binding sites (denoted by star symbols) reside on different aptamers and appear to be quite distant from each other. Interestingly, in 3D, a clear interaction path is visible between the two glycine binding sites, as seen in the difference map (Fig. 5C). From Apt-II, glycine binding leads to increased density for an adjacent Mg^2+^ connected to A206. This adenosine forms the conserved Hoogsteen base pair to U77 in Apt-I^23^, which is also stabilized by an attached Mg^2+^ bound to the Apt-I glycine. These data suggest a role for glycine where binding leads to stabilization of the fully folded tandem aptamer by rigidifying the inter-aptamer Hoogsteen base pair. It is notable that the magnesium density within the glycine binding pocket is either absent (as in Apt-II) or significantly diminished (as in Apt-I) in the absence of glycine (Fig. 5C), even though the magnesium concentration is held constant. Previous work has demonstrated that metabolites and cations bind cooperatively to riboswitches, where affinity is increased for the metabolite when cations are available, and vice versa^8^. While many studies describe cations as working as an ion cloud to offset the phosphate charges in the RNA backbone^30,31^, our data reveal that the glycine riboswitch also has two specific Mg^2+^ binding sites that synergistically interact with glycine within the binding pockets.

Crystallographic studies on individual glycine riboswitch aptamers indicate that glycine fits very snugly within the binding pocket and that the glycine itself is trapped by adjacent magnesium ions^24^. Our data similarly show compact binding pockets surrounded by several Mg^2+^ (Supplementary Fig. 7). In the Apt-I pocket, U80 and U81 undergo significant conformational transitions between the bound and unbound states (Fig. 5C and Supplementary Movie 3). If the nucleotide U80 were in its apo conformation, it would directly clash with bound glycine, thus necessitating this conformational shift. Additionally, the poorer density in this region in the absence of glycine suggests that these residues are more dynamic in the apo state. This conformational transition may therefore represent a potential pathway for glycine access to the binding pocket.

### Explicit solvent molecular dynamics simulations help validate glycine orientations

Previous crystallography studies on the *F. nucleatum* glycine riboswitch at 3.55 Å concluded that the Apt-II glycine is stabilized by direct interactions between the carboxyl group and the equivalent of U210, as well as the amino group and G176^23^. Although the 2.9 Å structure generated here is of insufficient resolution to confidently orient a small glycine ligand, our data are more consistent with an alternate arrangement where the nitrogen group points towards the adjacent phosphate from residue A175 (Supplementary Figs. 7 and 8). To distinguish between these two potential conformations, we performed explicit solvent all-atom molecular dynamics simulations using particle mesh Ewald electrostatics, with an environment of explicit bulk magnesium and potassium ions at 10 mM MgCl_2_ and 80 mM K^+^.

**Fig. 6:**
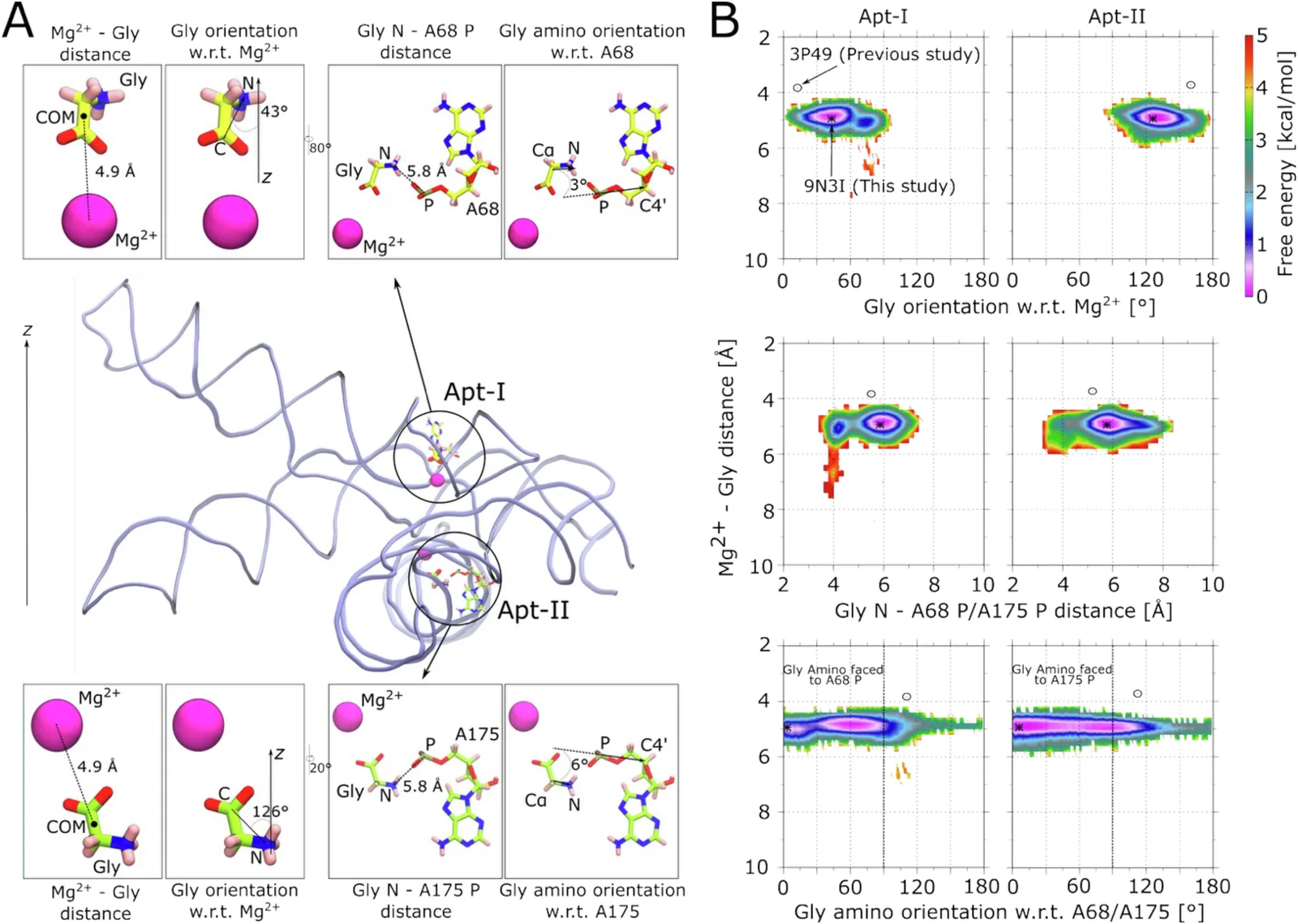
Glycine binding analysis using all-atom explicit solvent unbiased MD simulations. **A** To describe the binding of the glycine molecules, four variables were calculated for both aptamers: (i) distance between the Mg^2+^ and the center of mass of glycine, (ii) Glycine orientation, defined as the angle between the *z*-components of the vector passing from carboxyl C to amino N of glycine and the *z*-axis, (iii) distance between the amino N of the glycine and phosphate P of A68 of apt-I or P A175 of apt-II, and (iv) glycine amino orientation, defined as the angle between the vector passing from Cα to N of glycine and the vector connecting P and C4’ atoms of A68 or A175, where only *xy*-components of the vectors were utilized. Initial values of all variables for Apt-I and Apt-II glycines are mentioned on each panel. To facilitate both glycine orientations calculations, the whole riboswitch structure is aligned to the *z*-axis in the same manner as shown in the figure. **B** Free energy surfaces with respect to the Mg^2+^ -Gly distance and other variables, Gly orientation with respect to Mg^2+^, Gly N – A68 P /A175 P distance, and Gly amino orientation with respect to A68/A175. Surfaces on the left panels are for Apt-I, while on the right panels are for Apt-II. These surfaces are obtained by performing four 1-µs long equilibrium (unbiased) simulations. On all panels, the starting position of the glycines with respect to all variables are marked with the * symbol for our model (PDB: 9N3I), whereas with the ^⊙^ symbol for the previous model (PDB: 3P49).

First, using the unbiased simulations, we confirm that both glycines maintain a stable distance from the Mg^2+^ (~4.9 Å) and orientations with carboxyl groups facing toward the Mg^2+^ ions (Fig. 6B). In this position, the amino group of both glycines is seen to keep a stable distance (~5.8 Å) from the phosphate group of A68 (Apt-I) or A175 (Apt-II), and consistently maintain orientations faced towards these phosphate groups (Fig. 6B and Supplementary Fig. 8). The proposed orientation also puts the glycine amino group for Apt-II in feasible proximity of both the *N7* (3.4 Å N-N) and *O6* (2.9 Å O-N) of G176. Similarly, for Apt-I, the amino group is proximal to G69. Subsequently, enhanced-sampling explicit solvent metadynamics molecular dynamics simulations were carried out that sample the glycine molecules at all orientations. The resulting free energy surfaces show that when glycine maintains the 4 to 6 Å distance from the Mg^2+^ ion, the configurations with its carboxyl group facing the Mg^2+^ ion yielded the most favorable energy basins for both aptamers (Supplementary Fig. 8). At this favorable distance of glycines from Mg^2+^ ions, the glycine amino groups’ preferred position is around 5 to 7 Å away from the phosphate of A68 and A175, and they also have a preference to orient towards these phosphates (Supplementary Figs. 8 and 9), consistent with our proposed cryo-EM structure. Notably, four other potential metastable states seen for Apt-I pull the displaced glycine out of the corresponding experimental density (Supplementary Fig. 10). Both the explicit solvent molecular dynamics simulations and explicit solvent molecular dynamics simulations with metadynamics help to corroborate our proposed glycine orientations. These data also suggest that glycines bind to *V. cholerae* glycine riboswitch in an orientation that differs from previously described systems. Additionally, our binding pocket optimization yields a significantly improved conformation for the pocket when compared to the previously available, 5.7 Å resolution cryo-EM structure (PDB: 6WLU; Supplementary Fig. 5C)^33^, which lacked sufficient resolution to model the glycine. This workflow provides a means to combine mid-resolution cryo-EM data with MD to provide higher confidence about ligand-complex orientations.

## Discussion

Riboswitches play an important role in metabolism and regulate ~4% of bacterial genes in some species^38,39^. Riboswitch function is often described as regulation via alternation between two mutually exclusive conformations; however, this description belies the ensemble nature of biological macromolecules. In proteins, for example, conformational ensembles can be used to describe the mechanism of action for an enzyme or provide information about the biological role of disordered regions^40,41^. For RNA, an ensemble description is even more germane, as the significant flexibility inherent to the RNA backbone allows for the adoption of a greater variety of conformations^42–45^. NMR studies on the 2’ deoxyguanosine riboswitch have demonstrated that the RNA forms a varied set of ON, OFF, and intermediate conformations, even in the presence of ligand^46,47^. In fact, in vitro transcription experiments suggest that only 70% of the particles in some systems adopt a ligand-bound conformation in the presence of metabolite^46^. Riboswitch operation can thus be described by population states within an ensemble that shift in accordance with changing buffer conditions^48^. Here, cryo-EM explicitly captures this shift in populations and may, in future studies, illuminate the RNA folding pathway via characterization of low population states.

Our study shows that the glycine riboswitch adopts a highly dynamic “walking man” conformation, with several distinct stepping motions that coalesce in a well-resolved core surrounded by flexible loops and helices (Figs. 1 and 4). The lack of conservation in the dynamic regions (Supplementary Fig. 5)^21^ suggests that their role is not sequence specific. While it is not clear what role these variable regions play in riboswitch folding^23^, many studies have described a kinetic attribute in riboswitch regulation, where transcriptional pause sites provide time for the RNA structural rearrangements to mediate gene expression^46,47,49^. It is therefore possible that these flexible regions modulate the effect of ligand binding on expression levels by serving as an alternative to transcriptional pause sites, although this is currently speculative and will require future experimental validation.

Within each binding pocket, a coordinated Mg^2+^ is proximal to the bound glycine (Fig. 5). The marked reduction of magnesium density in the absence of glycine is notable. While this could indicate that this region is flexible in the absence of glycine, the local resolution of adjacent residues of this core region (Supplementary Fig. 3) suggests this region is relatively restricted. The data are instead more consistent with the absence or low localization of magnesium at these loci in this case. We therefore hypothesize that glycine stabilizes Mg^2+^ within the binding pocket, and that this interaction enhances glycine binding. In support of our conclusion, prior studies revealed a reciprocal cooperativity between ligands and Mg^2+^ for other riboswitches, with the presence of one binder increasing the affinity of the other^8^. Similar cation–ligand coupling has been observed or proposed in several other riboswitch systems. For example, Mg²⁺ concentration strongly affects ligand-induced folding of the TPP riboswitch, and structural studies of the glmS ribozyme show that the ligand is coordinated by hydrated Mg²⁺ ions within the active site, consistent with coupled metabolite and divalent-cation recognition^50–53^. This synergy has been seen for other cations too, such as with K⁺ enhancement of lysine affinity in the lysine riboswitch^54,55^. Additionally, one study used SAXS data (radius of gyration) to suggest that there are two specific divalent ion binding sites in the *V. cholerae* glycine riboswitch, which interact cooperatively with glycine to result in the final compact structure^32^. This series of RNA-ligand-cation interactions strongly stabilizes the core fold of the glycine riboswitch, suggesting an explanation for the larger question of why this riboswitch uses two adjacent aptamer domains. Based on our data, the presence of two aptamer domains helps the riboswitch achieve a more stable or robust structure. The linked domains may also provide structural redundancy, ensuring that the riboswitch folds correctly and functions efficiently across different environmental conditions.

This increased stability may be particularly important in the context of the full-length riboswitch, which includes the expression platform. Previous studies have suggested that switching behavior occurs between the terminator helix in the expression platform and the P1 helix of Aptamer-II^14,27^. In our suggested model, glycine binding would stabilize the inter-aptamer A-minor interaction between P3 of Aptamer-I and P1 of Aptamer-II (Fig. 2B), promoting formation of the terminator helix. On the other hand, under low glycine conditions, these inter-aptamer contacts are less frequently formed, potentially allowing for strand invasion and antiterminator helix formation. This shift in relative populations would favor transcriptional readthrough in the absence of glycine, resulting in increased gene expression from this OFF-type riboswitch. These structural findings are also consistent with prior chemical probing and mutational analyses of the *V. cholerae* glycine riboswitch, which identified critical nucleotides in the P1 and P3 helices and inter-aptamer junction as determinants of ligand affinity and cooperativity^22,56^. Our models reveal how these sites participate in a network of tertiary contacts (Supplementary Fig. 12) that couple glycine recognition to large-scale fold stabilization across the tandem aptamers. These structures provide an explanation for the mutational phenotypes observed in those earlier biochemical studies.

The modeled glycine orientation also provides a structural explanation for prior biochemical observations that certain glycine analogs, including glycine methyl ester and glycine tert-butyl ester, can be accommodated by the glycine riboswitch^14^, while other analogs like serine or sarcosine cannot. In our structures, the amino group and Cα position are tightly constrained by nearby nucleobase and backbone interactions, whereas the carboxylate-facing side of the ligand-binding pocket has comparatively greater available space (Supplementary Fig. 13). This geometry suggests that substitutions extending from the carboxyl group may be tolerated more readily than modifications near the amino group or Cα position, while still excluding many off-target amino acids or metabolites. Although our modeling of bulky glycine esters remains speculative, this pocket architecture is consistent with the observed ability of the riboswitch to bind specific glycine analogs while maintaining selectivity for glycine-like ligands.

Previous work on the *V. cholerae* glycine riboswitch has demonstrated that Apt-II binds glycine more tightly than Apt-I^24^. In addition, glycine binding by Apt-II is an enthalpically-driven reaction, while Apt-I interactions are entropically driven^24^, despite having nearly identical binding sites (Supplementary Fig. 7). These results suggest that more distal factors, such as the overall fold of the aptamer, influence the thermodynamics of glycine-aptamer interactions. Our ensemble analysis indicates that Apt-II is more stable than Apt-I in *V. cholerae* (Fig. 3 and Supplementary Fig. 1), potentially resulting from the extra G-C bond present within the active site (Supplementary Fig. 6). The difference in thermodynamic stability may therefore derive from additional folding garnered by glycine interactions with Apt-I, which are not required for Apt-II. Consistently, in some glycine riboswitch homologs, where Apt-I displays the higher affinity interactions, the extra G-C base pair is found in Apt-I instead of Apt-II^21^. We speculate that an ensemble analysis of these homologs would yield an inverse finding of aptamer stability, where the population of Apt-I is higher than Apt-II in the absence of glycine and/or Mg^2+^. Interestingly, several studies have shown that RNA folding and compaction increases as the concentration of Mg^2+^ increases^8,25,32,57^, raising the intriguing possibility of enriching partially folded conformations by sampling at various Mg^2+^ concentrations.

This study combines cryo-EM ensemble analysis with molecular dynamics simulations to identify ensemble shifts in response to ligand additions. However, several limitations should be considered when interpreting these in vitro ensembles. The RNA was refolded from a purified transcript under defined buffer conditions, which do not capture cellular crowding, the full complexity of physiological ion mixtures, or co-transcriptional folding by the polymerase. Because riboswitch regulatory decisions can be kinetically coupled to transcriptional pausing and nascent RNA folding, the populations observed here are best interpreted as equilibrium or near-equilibrium structural states of the isolated tandem aptamer construct rather than a complete description of the in vivo regulatory pathway.

Characterization of sparsely populated states, often the etiological agents of disease, has led to significant advancements in our understanding of misfolding-linked diseases^58,59^. Additionally, many disease-causing mutations operate at the ensemble level, where changes to the sequence result in abrogated populations of folds that can only be identified via an ensemble analysis^43^. Thus, it is important that work attempting to characterize RNA structure accounts for the highly heterogeneous nature of the biomolecule. Our work demonstrates that cryo-EM can be used to generate sub-3 Å RNA-only structures, provide information on RNA dynamics, and characterize population shifts in different conditions, allowing one to visualize the remodeling of the configurational landscape under various conditions. With the continuing advances in data collection and processing rates, it may soon be possible to capture mid to high-resolution information on RNA folding pathways and misfolds. While huge advancements in protein fold prediction have occurred since the release of AlphaFold^60^, the RNA field has less high-resolution training data available, not to mention the significant impact buffer conditions have on RNA folding^2^. A collection of large cryo-EM datasets may serve to address these challenges. The increasingly broad utilization of RNA within the fields of medicine and synthetic biology^61,62^ necessitates an improved understanding of RNA structure and dynamics.

## Methods

### RNA preparation

The *V. cholerae* gDNA template sequence used here (*GGCCTTCTAATACGACTCACTATAG*GTCCGTTGAAGACTGCAGGAGAGTGGTTGTTAACCAGATTTTAACATCTGAGCCAAATAACCCGCCGAAGAAGTAAATCTTTCAGGTGCATTATTCTTAGCCATATATTGGCAACGAATAAGCGAGGACTGTAGTTGGAGGAACCTCTGGAGAGAACCGTTTAATCGGTCGCCGAAGGAGCAAGCTCTGCGCATATGCAGAGTGAAACTCTCAGGCAAAAGGACAGAGGAG*TGAA*) includes both aptamer domains, but lacks the expression platform, and is based on the construct utilized in Kappel et al. (2020). The DNA template was generated by polymerase chain reaction using synthetic gblocks (IDT, Coralville, IA), with primers F: 5′-GGCCTTCTAATACGACTCACTATAGG-3′ and R: 5′-TTCACTCCTCTGTCCTTTTGCC-3′. The RNA was prepared through *in vitro* T7 RNA polymerase transcription using an AmpliScribe T7 High Yield Transcription Kit (LGC Biosearch Technologies; Hoddesdon, UK). The resulting RNA was then purified using RNAClean XP Beads (Beckman Coulter; Brea, CA) according to the manufacturer’s instructions and eluted in sterile Milli-Q water. Five separate preparations of RNA were combined and concentrated using a vacuum concentrator to generate the final 2.5 mg/ml sample. RNA purity was verified using both native and denaturing gel electrophoresis.

### Cryo-EM sample preparation and data collection

To compare the impact of Mg^2+^ and glycine, three RNA samples containing final solutions with (1) no ligands or cofactors, (2) 10 mM MgCl_2_, and (3) 10 mM MgCl_2_ plus 2 mM glycine (~150-fold the concentration of RNA) were prepared in parallel and treated equally. Purified RNA was refolded using a modified version of the protocol described previously^63^. Briefly, RNA solutions containing 1.2 mg/ml glycine riboswitch and 25 mM MES (pH 6.0) were denatured at 95 ^o^C for 4 min and flash cooled on ice for 5 min. Either MgCl_2_ or MgCl_2_ and glycine were added to respective samples, and all three RNA solutions were then incubated at 37 ^o^C for 30 min. Samples were stored at 4 ^o^C until grids were prepared (~1 h).

Refolded RNAs were applied as 3.5 μl aliquots to Quantifoil R1.2/1.3 300-mesh gold grids (EM Sciences, Prod. No. Q350AR1.3). Grids were glow-discharged with a Gatan Solarus instrument for 30 s at 15 mA before sample application. Grids were blotted for 3 s in an FEI Vitrobot Mark IV (Thermo Fisher Scientific), set to 4 °C and 100% humidity, prior to plunge-freezing into liquid ethane.

Cryo-EM data were collected at the Columbia University Cryo-Electron Microscopy Center (CEC). Data were collected with a pixel size of 0.823 Å on a Titan Krios G3i (Thermo Fisher Scientific) operated at 300 kV using a Gatan K3 BioQuantum direct electron detector. A total of three Leginon^64^ data collections were used to generate the structures for the glycine riboswitch without MgCl_2_ or glycine, with 10 mM MgCl_2_, and with 10 mM MgCl_2_ and 2 mM glycine. Data collection statistics are summarized in Supplementary Table 1.

### Cryo-EM data processing

The three datasets were processed using cryoSPARC (v4.6.0)^36^. The procedure is outlined in Supplementary Fig. 1. Briefly, in all datasets, movie alignments, drift correction, and dose weighting were done using cryoSPARC’s patched implementations. Micrographs with poor CTF fits or non-ideal ice thickness were removed, resulting in 5338 micrographs for the holo dataset, 5819 micrographs for the MgCl_2_ dataset, and 4312 micrographs for the no-MgCl_2_, no-glycine dataset (Supplementary Table 1). For all datasets, particles were first auto-picked using cryoSPARC’s blob picker job to select particles with dimensions from 70 to 120 Å in an unbiased manner. Particles were then extracted with a box size of 256 px (210 Å), and datasets were cleaned using iterative 2D classification to remove obvious junk/spurious classes while conserving the heterogeneity in the sample (Supplementary Fig. 2). For each sample, ten ab initio models were generated from cleaned particle pools to characterize how ligands and cofactors impact structural heterogeneity. Structures were then heterogeneously refined and aligned using cryoSPARC’s “Align 3D” job (Fig. 3 and Supplementary Fig. 1)^36^.

To generate final maps for the MgCl_2_ and holo samples, 3D classes consistent with the fully folded construct were selected and filtered via successive rounds of 3D classification. Final datasets were obtained via reference-based motion correction, and non-uniformly refined^65^ without adaptive marginalization or dynamic masking to produce the final maps. Map resolution was determined by the Fourier shell correlation (FSC) between two half-maps at a value of 0.143 with FSC-mask auto-tightening, resulting in ostensible resolutions of 2.9 Å (215,269 particles) for the holo sample, and 3.3 Å for the MgCl_2_ sample. To better understand the dynamic motions present in the holo complex, the final particle pool was 3D-classified into four maps, using a filter resolution of 5 Å in the cryoSPARC 3D classification job (Fig. 4)^36^. Structural heterogeneity was further explored via 3D-variability analysis with 20 models at filter resolutions of 5 Å (Supplementary Movies 1 and 2)^37^. Although attempts were made to further refine and resolve the leg regions using various cryo-EM toolkits that focus on dynamic regions, such as cryoDRGN^66^, 3DFlex^67^, DynaMight^68^, or multibody refinement, we were unable to obtain a high-resolution reconstruction of the leg regions.

### Model building and refinement

A mid-resolution structure of the *V. cholerae* glycine riboswitch is available (PDB-6WLT)^33^, which was used as the initial template for modeling our structures in Coot (v.9.8.95)^69^. RNA A-form restraints were used extensively to ensure low-resolution areas did not collapse during refinement. Model geometries and fits to maps were adjusted in ISOLDE (v1.8)^70^ to decrease issues arising from steric clashes, with restraints placed on RNA base pairs. Low-resolution regions in the “legs” and Apt-II P3 were refined in a filtered map to ensure backbone placement was reasonable, but care should be taken to interpret finer details in any of these low-resolution regions (Supplementary Fig. 3). Mg^2+^ was only modeled when there was either sufficient biochemical evidence (such as coordination to phosphate groups within the glycine binding site), or if information could be derived from homologous regions of a previously available 3.55 Å crystal structure from the related *Fusobacterium nucleatum* glycine riboswitch (PDB-3P49)^23^. Where neither method was applicable, ligands were left unmodelled to avoid overinterpreting the data. Final refinements were performed using PHENIX (v1.21.1-5286-000)^71^ real space refinement against the final maps for both the holo and MgCl_2_ samples. Model statistics are disclosed in Supplementary Table 1. Structures and maps were visualized and presented using ChimeraX^72^. The 3D structure was used to determine structural elements and contacts, which were then depicted using RNAscape^73^. Long-range inter- and intra-aptamer contacts were indicated with Leontis–Westhof symbols^74^ where the interaction geometry could be assigned from the model and map (Supplementary Fig. 12).

### Explicit solvent molecular dynamics simulations

The structure of the glycine riboswitch obtained in the 10 mM MgCl_2_ plus 2 mM glycine condition was used to perform all-atom explicit solvent molecular dynamics simulations. The riboswitch structure was solvated with the TIP3P water molecules and 10 mM MgCl_2_. Notably, a bulk concentration of 10 mM Mg^2+^ ions (bulk is defined by the region >12 Å away from the RNA) was achieved following conducting several cycles of equilibration stages using the protocol described previously^75^. Moreover, 140 mM K^+^ ions were added for neutralizing the whole system to net charge zero, which resulted in a bulk concentration of 80 mM K^+^. The resulting systems had approximately 240,000 atoms in total. The amber ff99bsc0χ_OL3_ force field for RNA^76^, amber ff19SB for zwitterionic glycine^77^, and recently optimized parameters for the ions Mg^2+^, K^+^, and Cl^-^ with TIP3P water were used^78,79^. The cut-off for short-range electrostatics and the Van der Waals interactions was 12 Å, and the PME method^80^ was used for the long-range electrostatics with a grid size of 1.2 Å. Hydrogen-containing bonds were constrained using the LINCS algorithm to enable a timestep of 2 fs^81^. To check the stability of glycines in Apt-I and Apt-II in our proposed orientations, we used four unbiased simulations for a timescale of 1 µs. All MD simulations were carried out using the GROMACS v2024.3^82^ patched with PLUMED v2.9.2^83^.

### Metadynamics explicit solvent molecular dynamics simulations

To further support our proposed glycine orientations, we employed the combined well-tempered and multiple walker metadynamics technique^84,85^. To enforce reorientation of the glycine in both aptamers, two collective variables for Apt-I and Apt-II are defined as the angle between the *z*-components of the vector passing from carboxyl carbon to amino nitrogen atoms of a glycine molecule and *z*-axis^86^. To define these collective variables, the glycine molecules were aligned with respect to the *z*-axis as shown in Fig. 6A, and the positions of all the phosphorus atoms of RNA were restrained during the simulations to maintain the alignment of the whole riboswitch in that fashion. Three independent simulations for a total timescale of 2 µs were carried out. In each simulation, two metadynamics runs, each having eight walkers, were performed simultaneously by biasing the angle collective variables defined for Apt-I and Apt-II^87^. Along the collective variable, Gaussians with the height of 1 kJ/mol and width of 1° were deposited at the interval of 2 ps. The bias factor was set to 15, corresponding to a tuning temperature of 4200 K. Upon completion of simulations, two-dimensional free energy surfaces as a function of collective variables, distance between Mg^2+^ and center of mass of glycine and the angle of glycine with respect to the z-axis, were calculated using the Tiwary–Parrinello reweighting scheme^88^.

### Flexible simulations using SMOG

The initial cryo-EM structure was used to generate the SMOG native contact potential^89^. All simulations were performed using the MD software package GROMACS^90^. The initial configuration and topology files for GROMACS were generated using the SMOG web tool^91^. The starting structure was minimized for 10,000 steps using the SMOG native contact potential. MDFIT^92,93^ and cryo_fit^45^ were employed for flexible fitting into the generated cryo-EM map. To accurately match the cryo-EM density map with the configurational map, a 10^5^-step MD simulation was conducted to evolve the simulation towards a stable structure. Principal component analysis (PCA) was performed on the MD trajectories to gain insights into collective motion. The inbuilt subroutine, gmx covar, in GROMACS was used to generate both eigenvalues and eigenvectors, followed by filtering the individual eigenvectors to analyze the two largest eigenvalues. For visualization, VMD^94,95^ and UCSF Chimera were utilized.

## Supplementary information

Supplementary information

Supplementary Movie 1

Supplementary Movie 2

Supplementary Movie 3

Supplementary Movie 4

## Data Availability

The cryo-EM density maps and model coordinates have been deposited in the EM Data Bank (https://www.ebi.ac.uk/pdbe/emdb/) and the Protein Data Bank (https://www.rcsb.org) with accession codes EMD-48851 and 9N3I for the holo glycine riboswitch sample, and EMD-48852 and 9N3J for the Mg^2+^ alone sample. A previously published model by Kappel et al. (2020) was used as a starting template (PDB code: 6WLT)^33^.
