# Supplementary material for: Structural basis for Mg²⁺–glycine synergy in the tandem glycine riboswitch revealed by cryo-EM
Source: Npj Struct Biol. 2026 Oct 1;1(1):2. doi: 10.1038/s44511-026-00002-y (PMC13648278; doi:10.1038/s44511-026-00002-y)
Supplement: Supplementary file 1 [file 44511_2026_2_MOESM1_ESM.pdf]

Supplementary Information to:

Structural Basis for Mg^{2+}–Glycine Synergy in the Tandem Glycine Riboswitch Revealed by Cryo-EM

Nathan Jespersen[1], Jigneshkumar Dahyabhai Prajapati[1], Ankush Singhal[1], Karissa Y. Sanbonmatsu[1,2*]

[1]Theoretical Biology and Biophysics, Los Alamos National Laboratory, Los Alamos, NM 87545, United States

[2] New Mexico Consortium, Los Alamos, NM 87544, United States

*Corresponding Author: Karissa Y. Sanbonmatsu - kys@lanl.gov

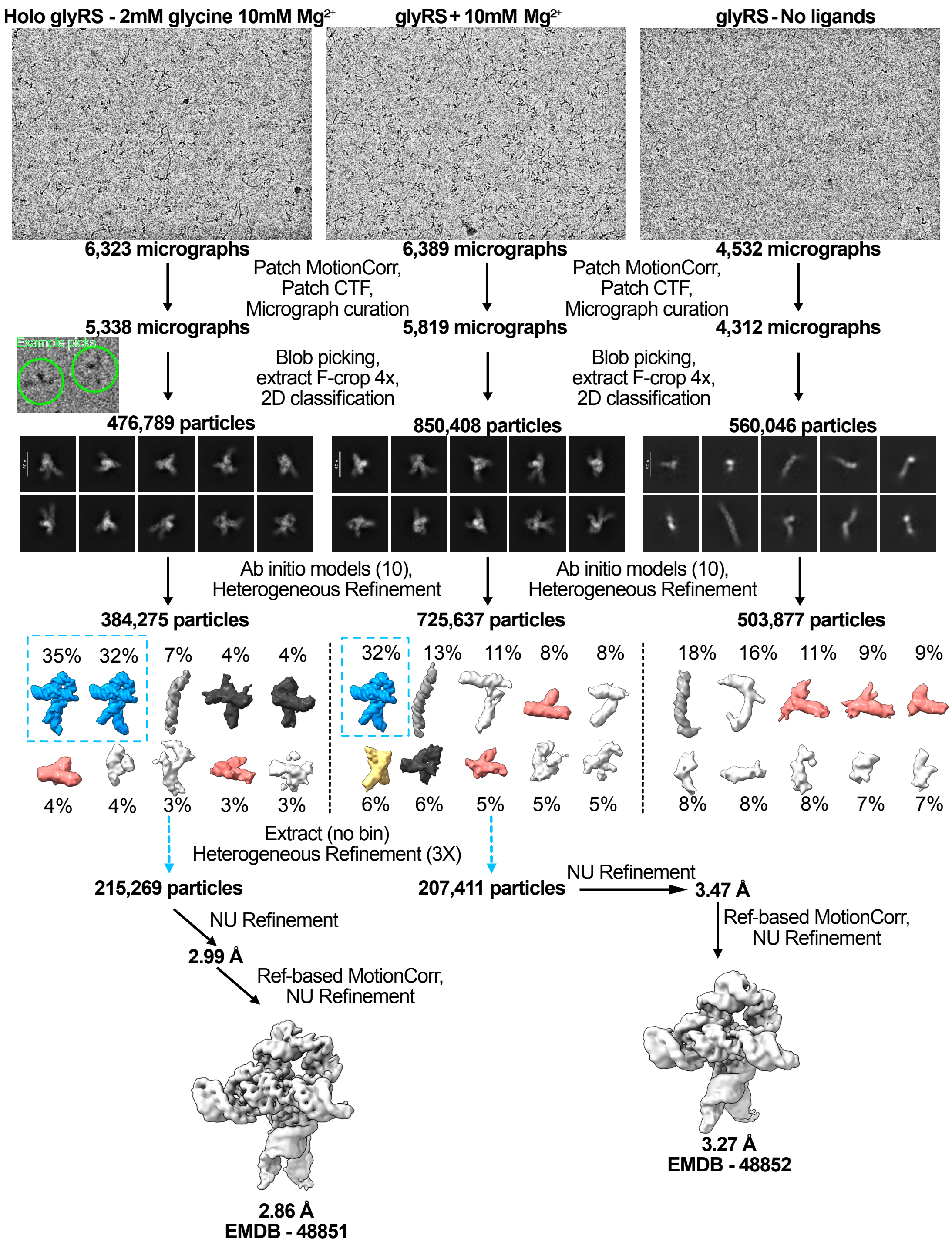

Supplementary Fig. 1: Cryo-EM data processing scheme. Data collection and processing procedures for the three glycine riboswitch datasets. From left to right: holo (2 mM glycine, 10 mM Mg^{2+}), apo (no glycine, 10mM Mg^{2+}), and neither glycine nor Mg^{2+}. Volume colors correlate to maps that align well and may represent conserved fold for walking man (blue), a long helix (grey), Apt-II (pink), Apt-I (black), and the core (black).

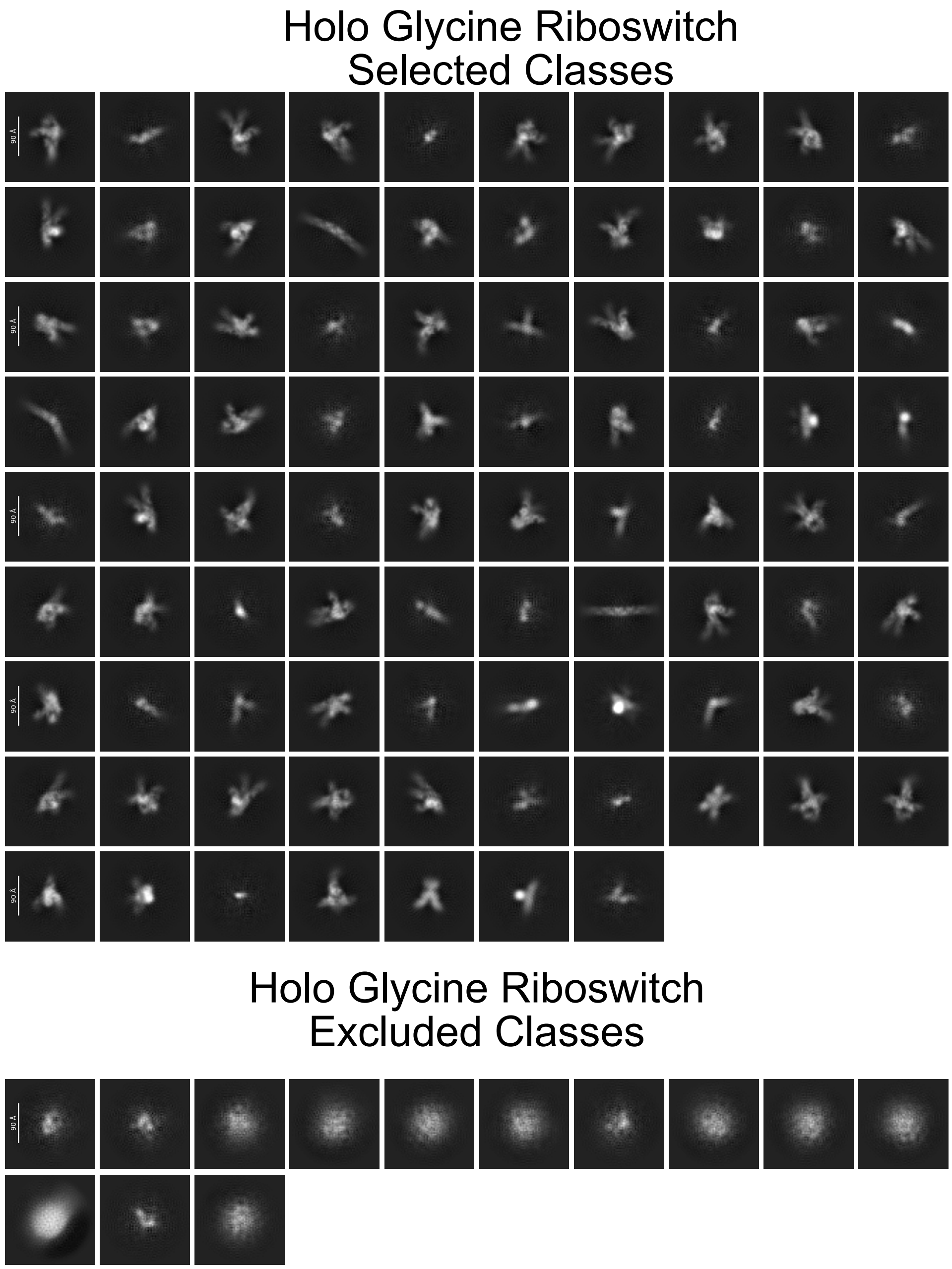

Supplementary Fig. 2: 2D classes selected or excluded in classification for the holo glycine riboswitch. In total, 87 classes (478,626 particles) were selected, and 13 classes (67,151 particles) were excluded due to low feature signal.

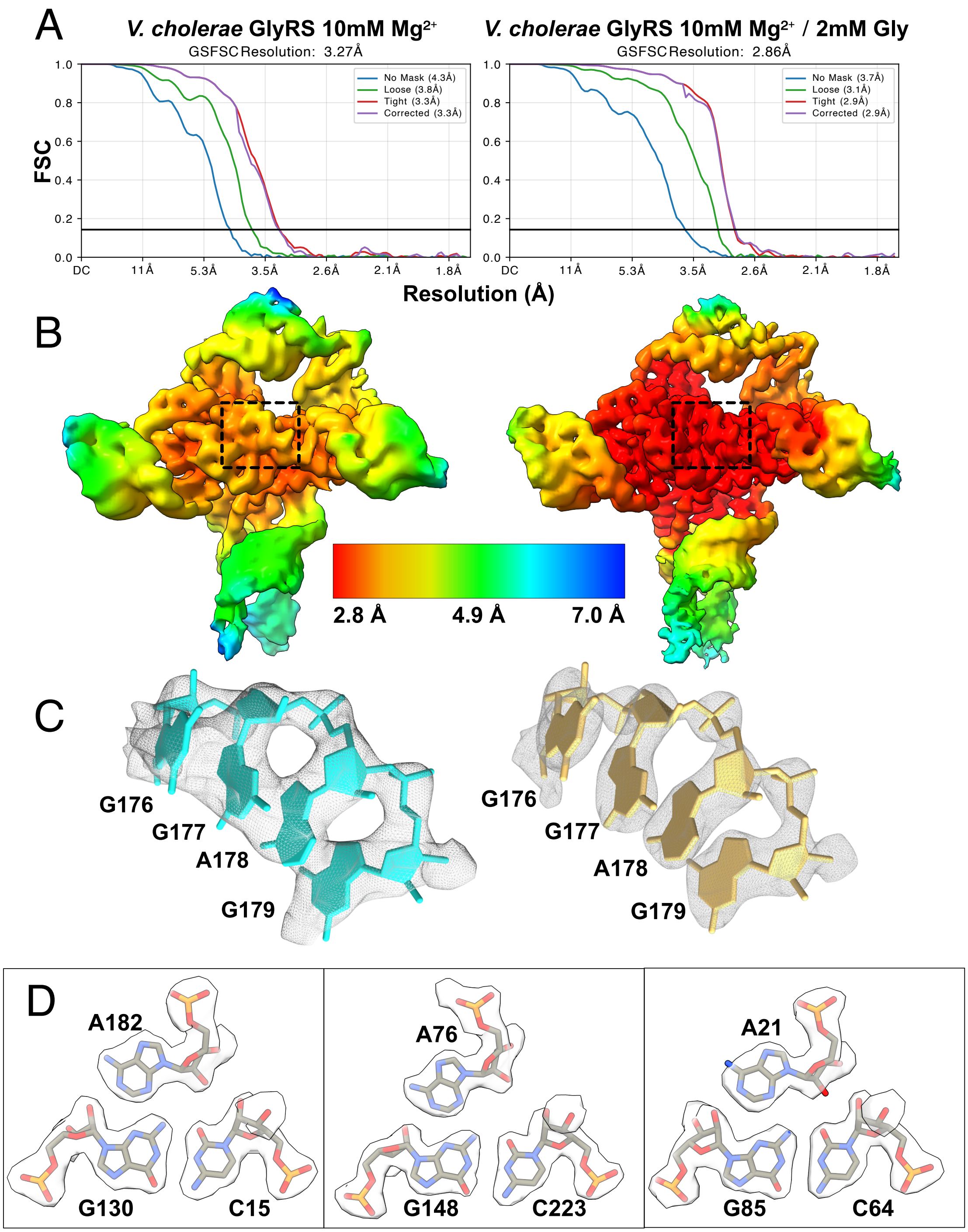

Supplementary Fig. 3: Overall and local resolution estimation. a Fourier Shell Correlation (FSC) plot generated in cryoSPARC for the holo (right) and apo (left) glycine riboswitch structures. **b** Surface representations of the respective maps colored based on local resolution. **c** Expanded view of the boxed region in *b*, showing the density and model fit in the core region. **d** Example images of three A-minor motifs present within the structure and their resolvability.

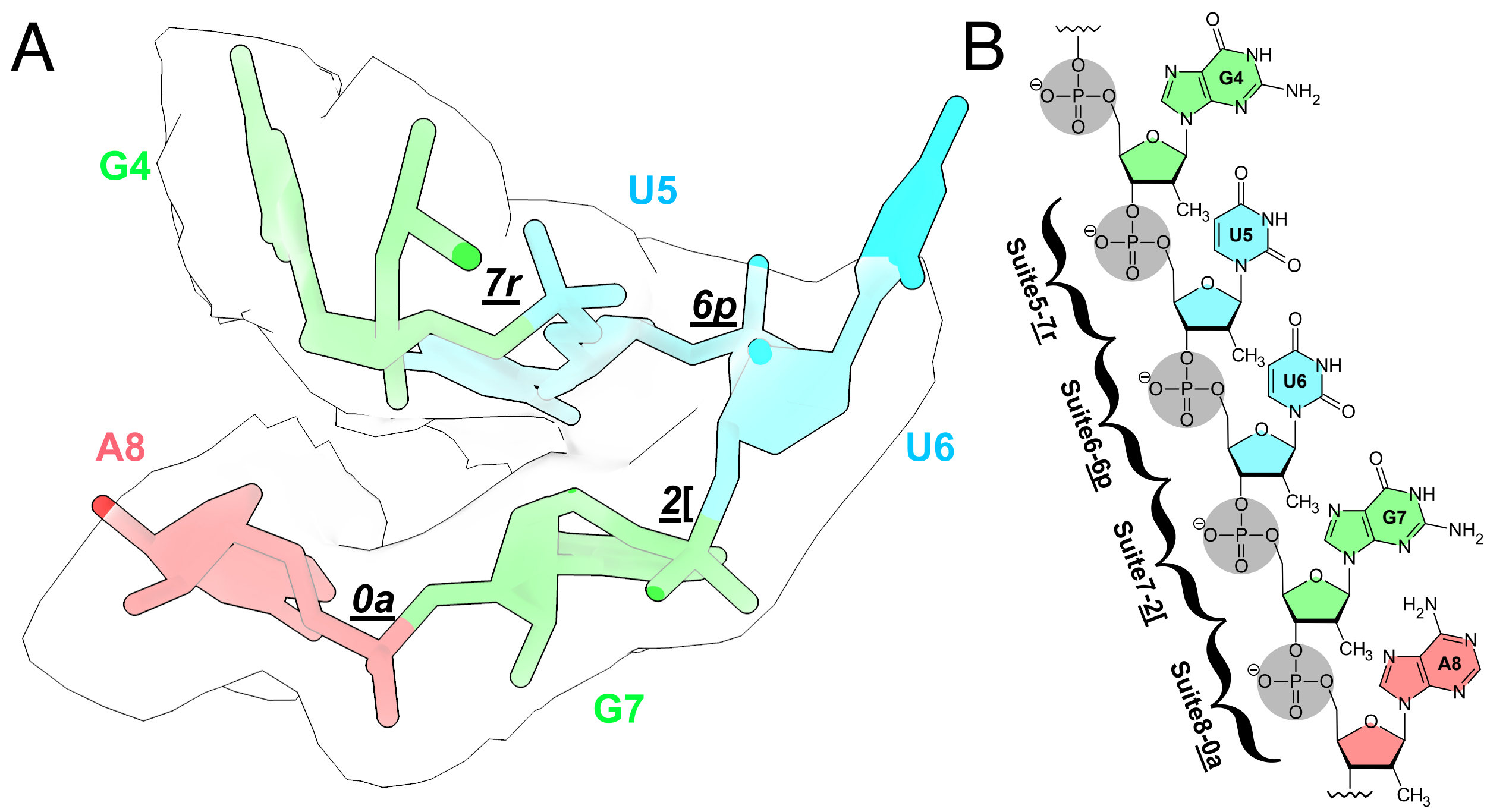

Supplementary Fig. 4: The P0 region of the glycine riboswitch adopts RNA suites consistent with a kink-turn conformation. a Overlay of residues 4-8 in the holo glycine riboswitch structure with a transparent density map. Colors correspond to base identity. Suites for each sugar-to-sugar set of torsion angles are included. **b** Chemical view of each nucleotide and suite in the kink-turn.

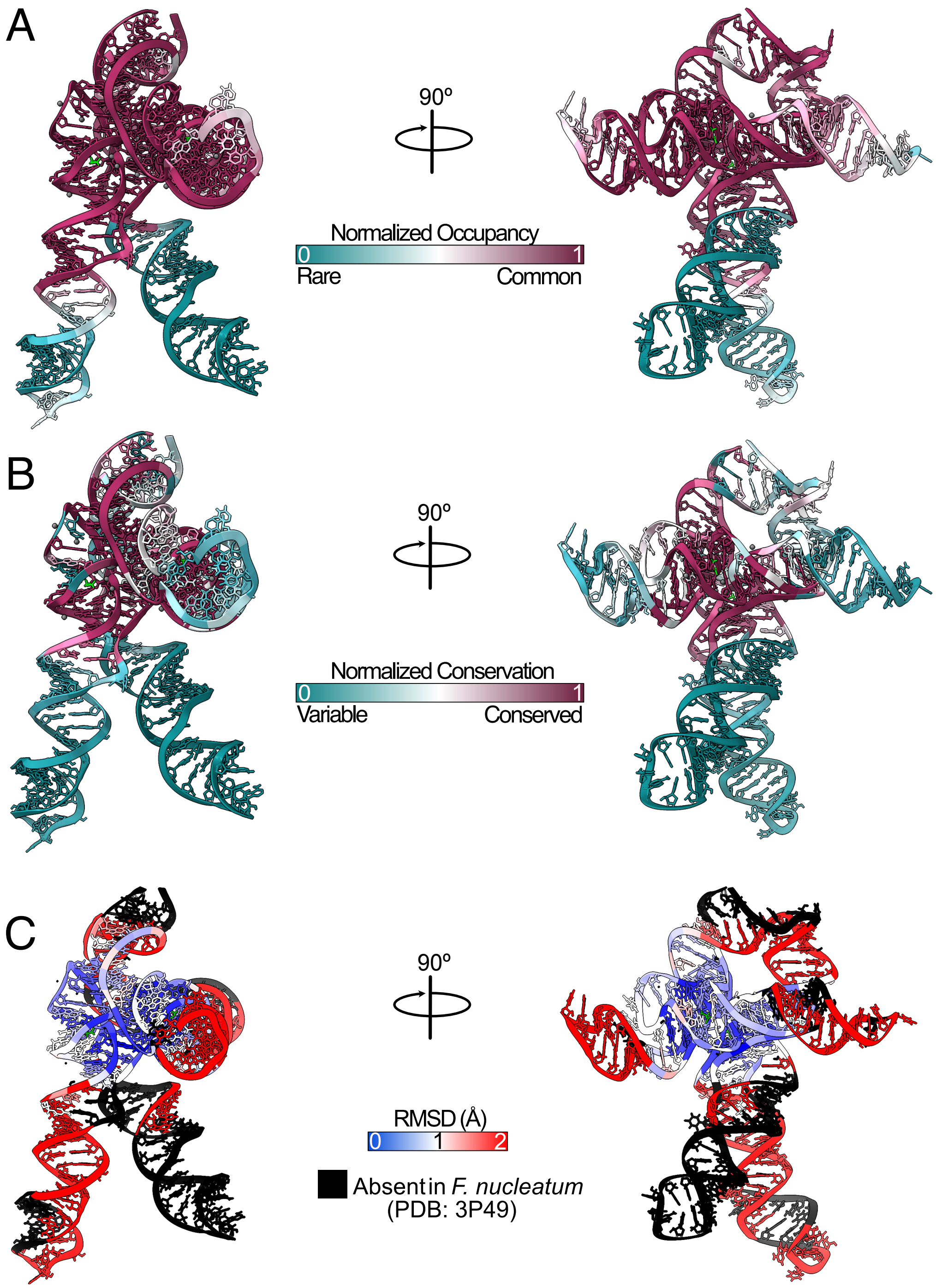

Supplementary Fig. 5: The glycine riboswitch core is conserved in both sequence and structure. a Normalized occupancy of each residue within the *V. cholerae* glycine riboswitch across all identified homologs. **b** Normalized conservation for each residue within the *V. cholerae* glycine riboswitch across all identified homologs. Normalized values were calculated with gaps included. Values from *a,b* were calculated using 11,626 glycine riboswitch sequences identified, and alignments made, in Torgerson *et al*. (2020)[1]**. c** RMSD calculated in ChimeraX[2] using the *F. nucleatum* glycine riboswitch (PDB code: 3P49)[3] and the *V. cholerae* structure solved here. Regions colored in black are absent in *F. nucleatum*.

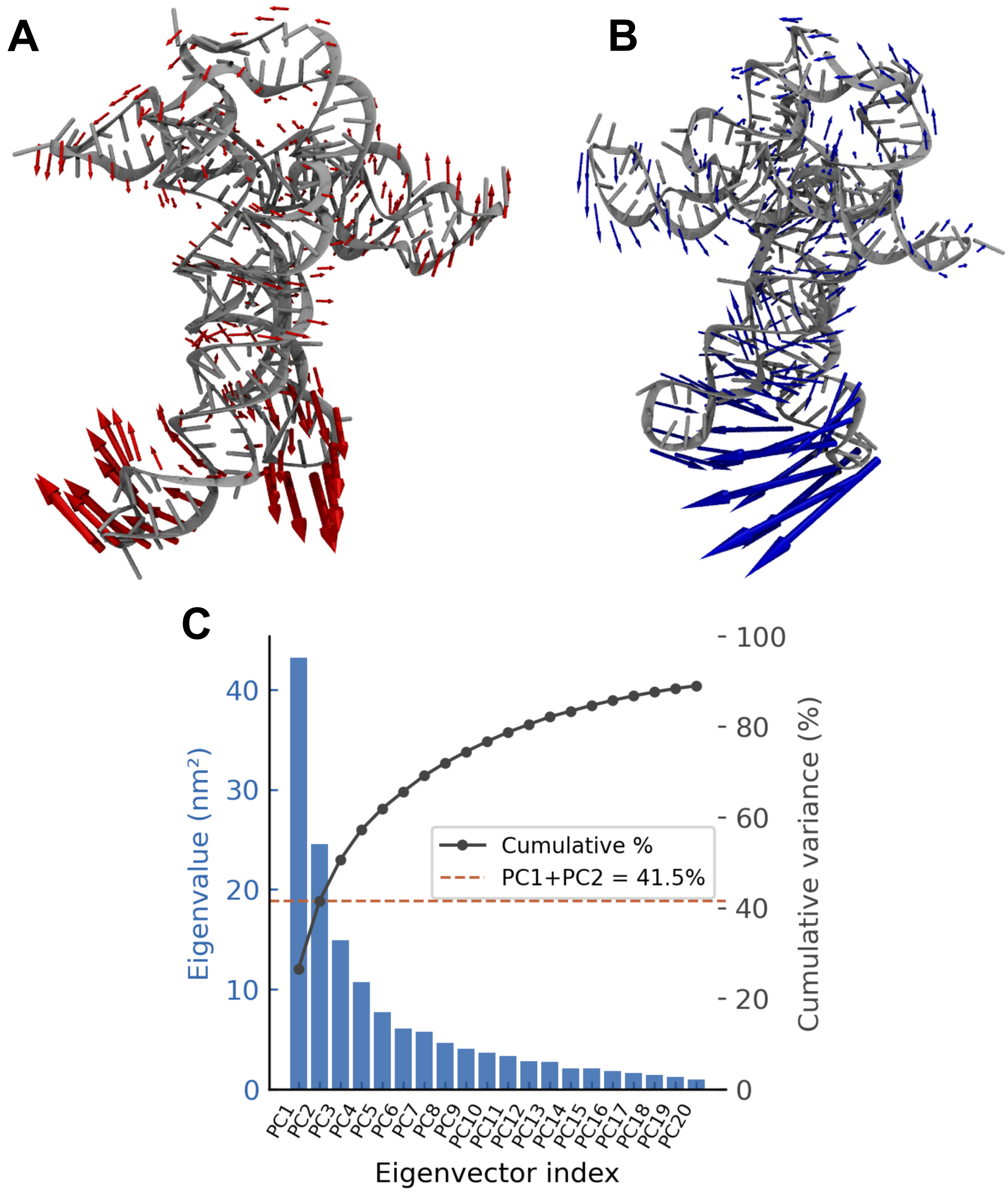

Supplementary Fig. 6: Dominant principal-components of the glycine riboswitch motions. Arrows are drawn at the phosphorus atom of each nucleotide and represent the direction and relative magnitude of motion along (a) PC1 (red) and (b) PC2 (blue), as derived from the eigenvectors of the atomic covariance matrix computed over the SMOG simulation. Arrow length and shaft thickness scale linearly with displacement magnitude. The average structure is shown as a grey cartoon. **c** Bar heights (left axis, blue) represent the eigenvalues of the covariance matrix for the top 20 eigenvectors, reflecting the configurational variance captured by each principal component. PC1 and PC2 account for 41.5% of the total variance (dashed red line), indicating these two modes capture some of the dominant collective motions of the riboswitch.

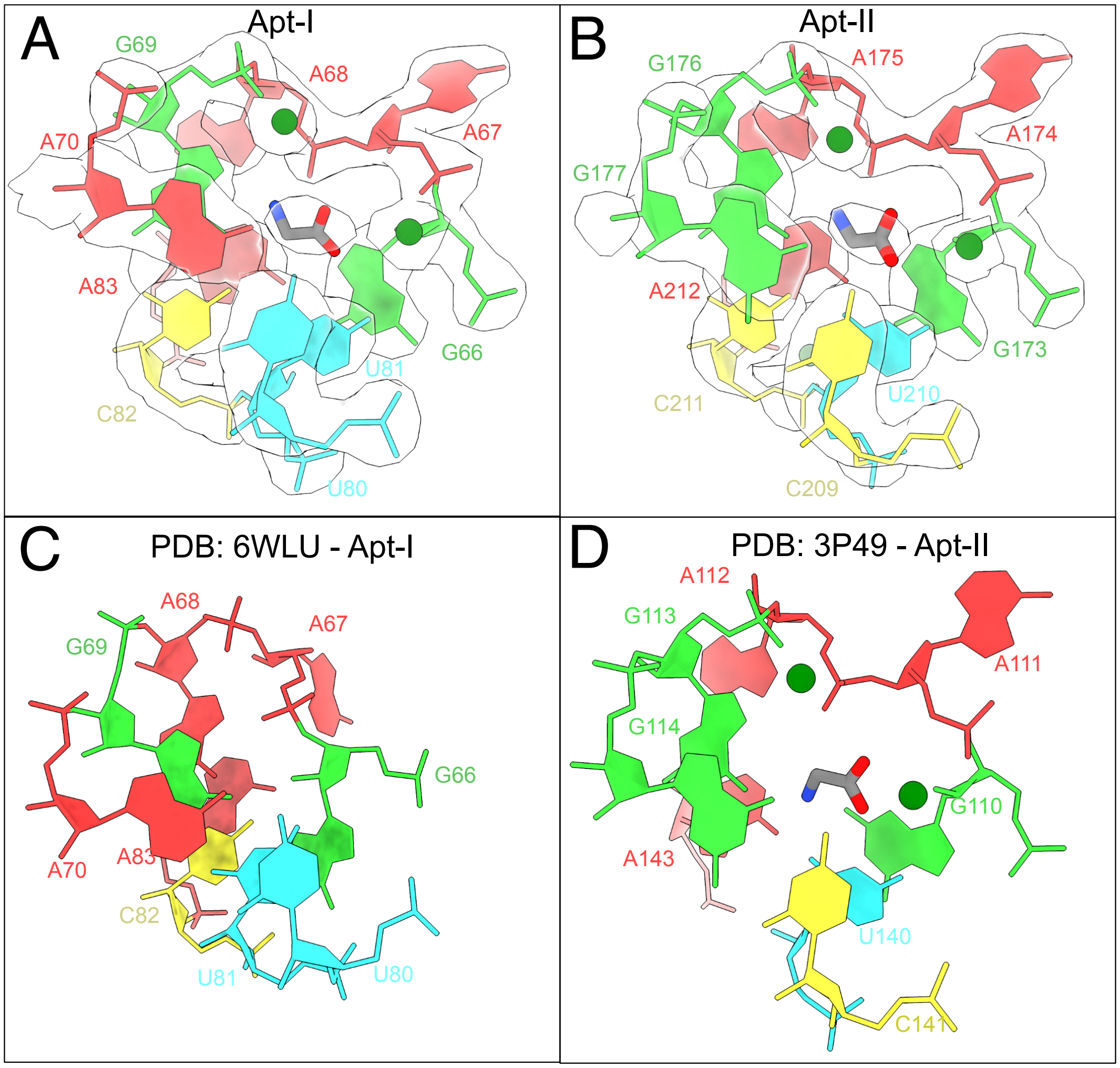

Supplementary Fig. 7: Comparison of glycine riboswitch binding pockets. Residues within 4 Å of the bound glycine are shown for the *V. cholerae* glycine riboswitch Apt-I (a) and Apt-II (b) from this publication, a previously published Holo Apt-I from *V. cholerae* (c; PDB: 6WLU)[4], and *F. nucleatum* (d; PDB: 3P49)[3]. Glycines are colored with carbons in grey and magnesium ions proximal to the binding site are shown and colored dark green. Nucleotides are colored according to base identity. Binding sites between *V. cholerae* aptamers are largely identical, with the exception of the A70-U80 (Apt-I) to G177-C209 (Apt-II) change. Significant improvements have been made in geometry of the glycine binding pocket compared to previous publications (a vs c). The published *F. nucleatum* PDB displays an alternative glycine conformation in which the nitrogen is rotated towards U140.

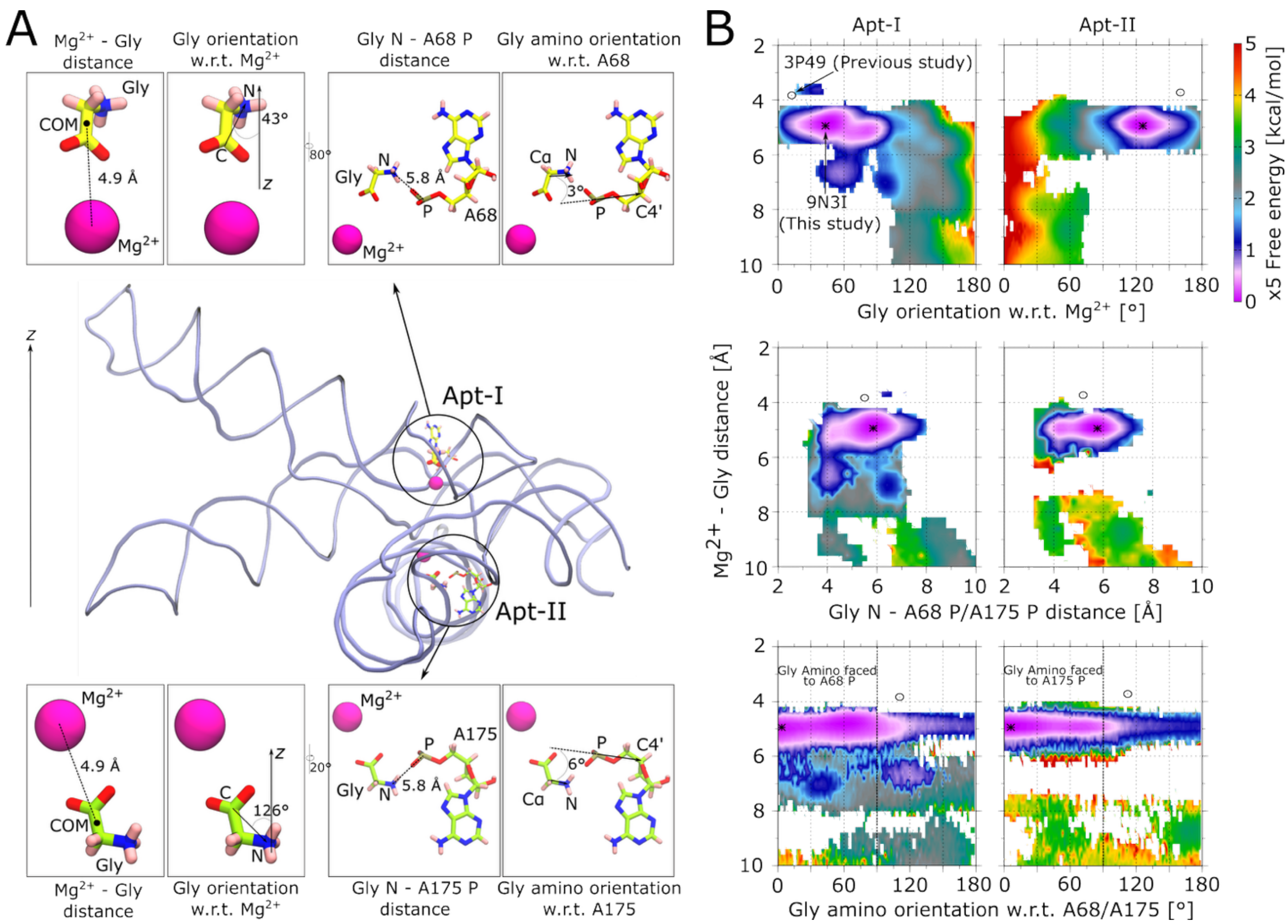

Supplementary Fig. 8: Glycine binding analysis using all-atom explicit solvent metadynamics simulations. a To describe the binding of the glycine molecules, four variables were calculated for both aptamers: i) distance between the Mg^{2+} and the center of mass of glycine, ii) Glycine orientation, defined as the angle between the *z*-components of the vector passing from carboxyl C to amino N of glycine and the *z*-axis, iii) distance between the amino N of the glycine and phosphate P of A68 of apt-I or P A175 of apt-II, and iv) glycine amino orientation, defined as the angle between the vector passing from Cα to N of glycine and the vector connecting P and C4' atoms of A68 or A175, where only *xy*-components of the vectors were utilized. Initial values of all variables for Apt-I and Apt-II glycines are mentioned on each panel. To facilitate both glycine orientations calculations, the whole riboswitch structure is aligned to *z*-axis in the same manner as shown in the figure. **b** Free energy surfaces with respect to the Mg^{2+} -Gly distance and other variables, Gly orientation with respect to Mg^{2+}, Gly N – A68 P /A175 P distance, and Gly amino orientation with respect to A68/A175. Surfaces on left panels are for Apt-I while on the right panels are for Apt-II. These surfaces are obtained by performing three 2 µs long metadynamics simulations, where the variable Gly orientation with respect to Mg^{2+} for both glycines were biased to reorient glycines in all possible orientations. In all three simulations, excellent convergence in the free energy surfaces were observed (Supplementary Figure 9). On all panels, the starting position of the glycines with respect to all variables are marked with the * symbol for our model (PDB: 9N3I), whereas with the ⊙ symbol for the previous model (PDB: 3P49).

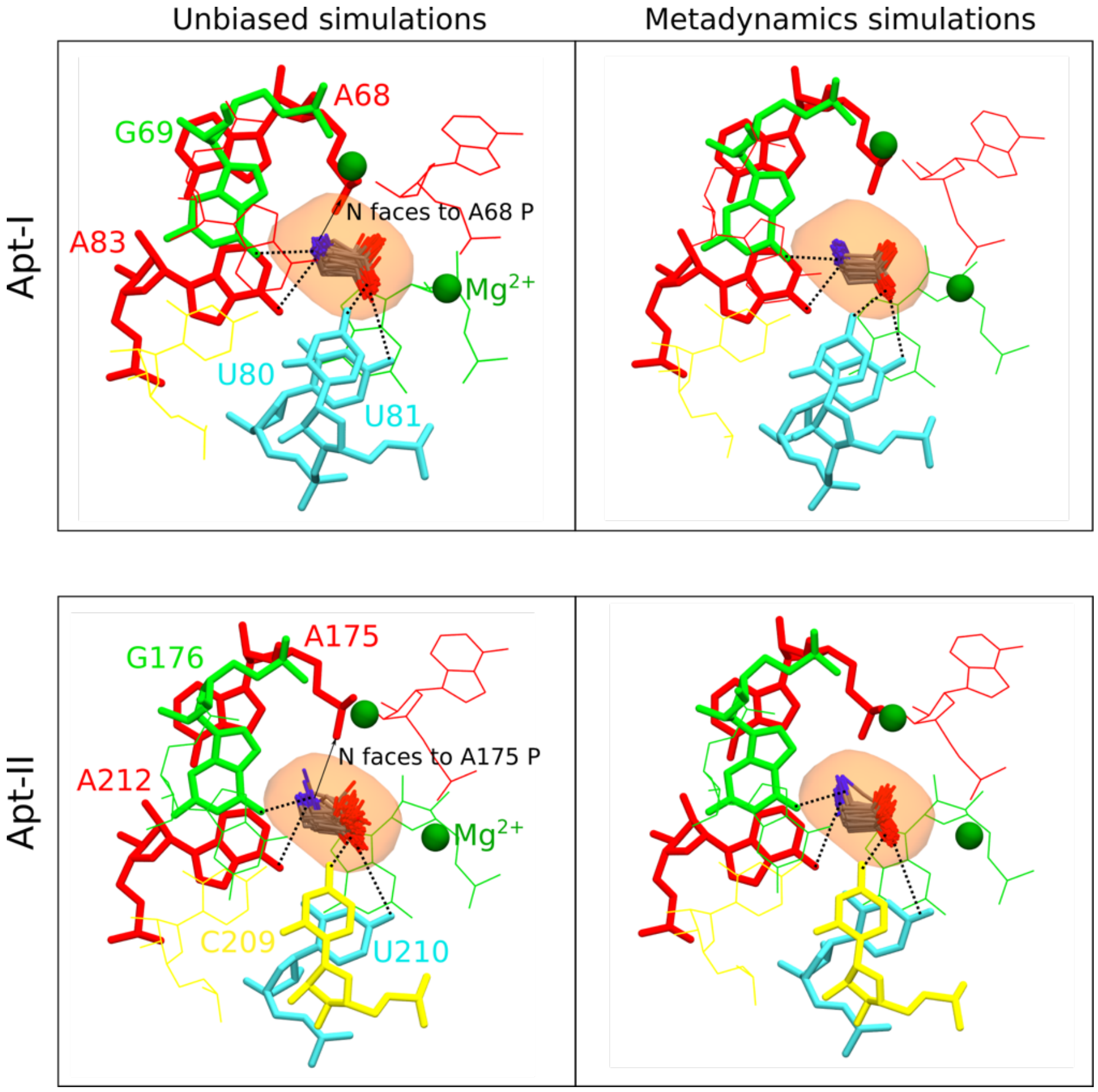

Supplementary Fig. 9: Glycines remain stable in our proposed configurations for Apt-I and Apt-II. From unbiased and metadynamics simulations trajectories, several thousand frames representing the initial state conformations (Fig. 6) are extracted, and approximately one hundred conformations are depicted for glycine in each aptamer. Glycines maintain constant distance from the Mg^{2+} ions, with the carboxyl group facing toward the Mg^{+2} ions and the amino group oriented toward the phosphate group of A68 and A175 in Apt-I and Apt-II, respectively, indicating our proposed glycine configurations are most stable.

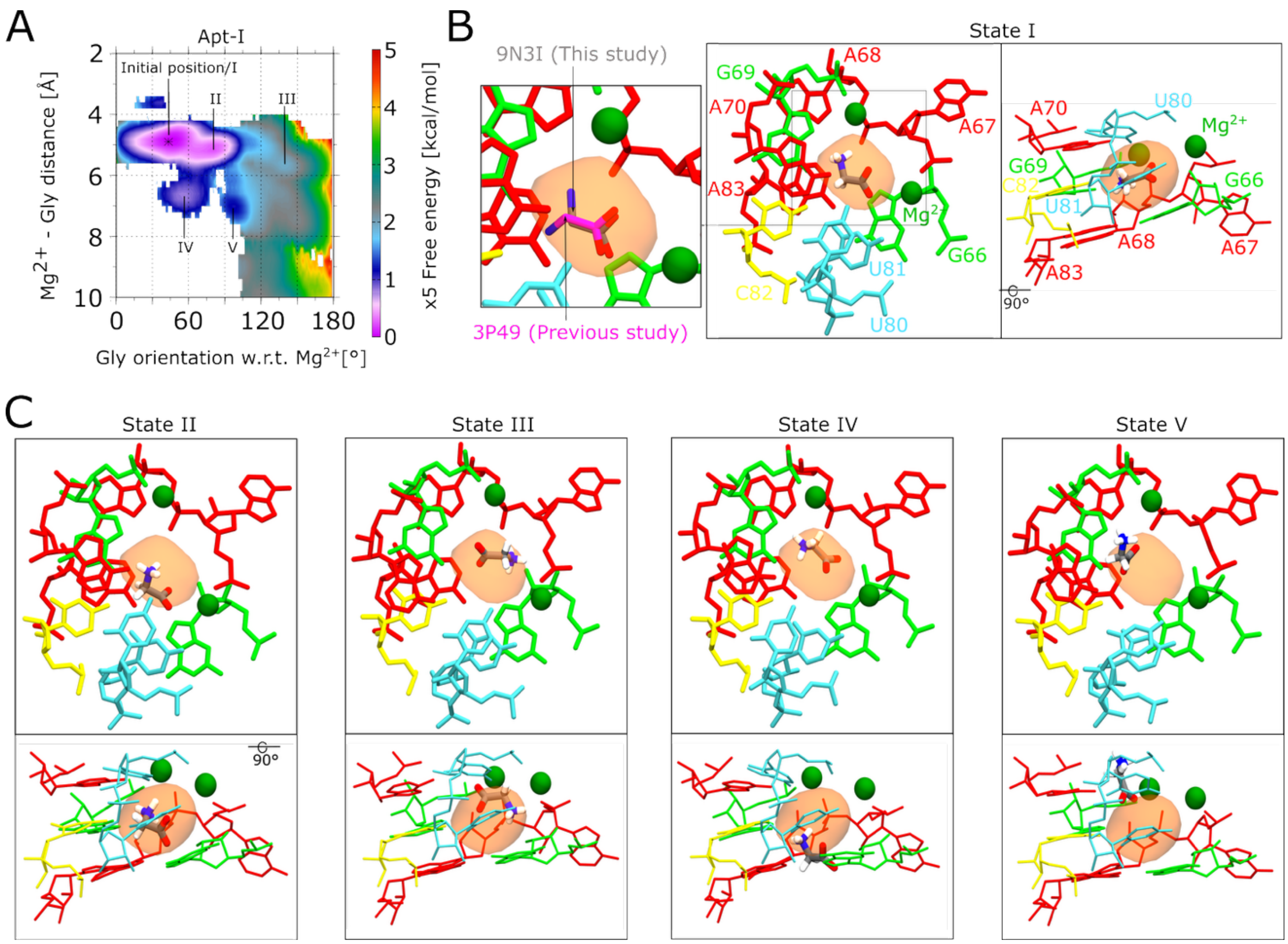

Supplementary Fig. 10: Glycine conformations in metastable states seen in vicinity of modeled state. a Free energy surfaces with respect to the Mg^{2+}-glycine distance and glycine orientation for Apt-I (consistent with Fig. 6b). Five minima over the free energy surface, including a minimum representing the initial state of glycine modeled from the cryo-EM map, denoted as state I, are marked. **b** Conformation of glycine in minimum I, along with the neighboring residues, Mg^{2+} ions and cryo-EM-derived, low-pass filtered glycine density, are shown from two perspectives. On the left panel, the conformations of the glycine from this study (minimum 1) and from the previously reported structure are compared (3P49). **c** Similarly, glycine conformations in four additional minima are illustrated.

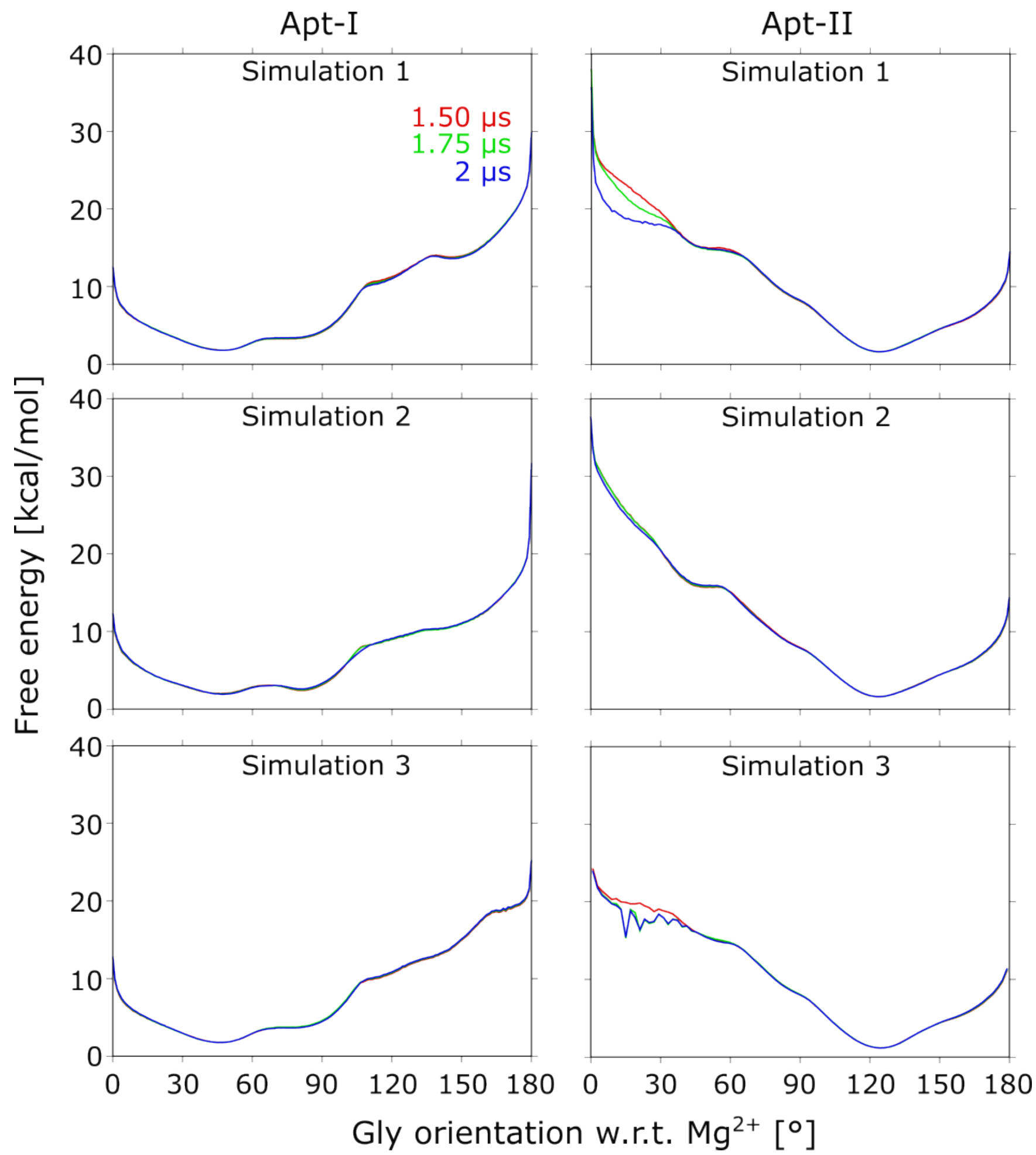

Supplementary Fig. 11: Convergence in metadynamics simulations. Free energy surfaces with respect to the variable Gly orientation with respect to Mg^{2+} are shown for all three simulations at timescales of 1.5,1.75, and 2 μs for Apt-I (left panels) and apt-II (right panels). Very minimal variations in the free energy profiles were observed from 1.5 to 2 μs, indicating excellent convergence was achieved in all metadynamics simulations.

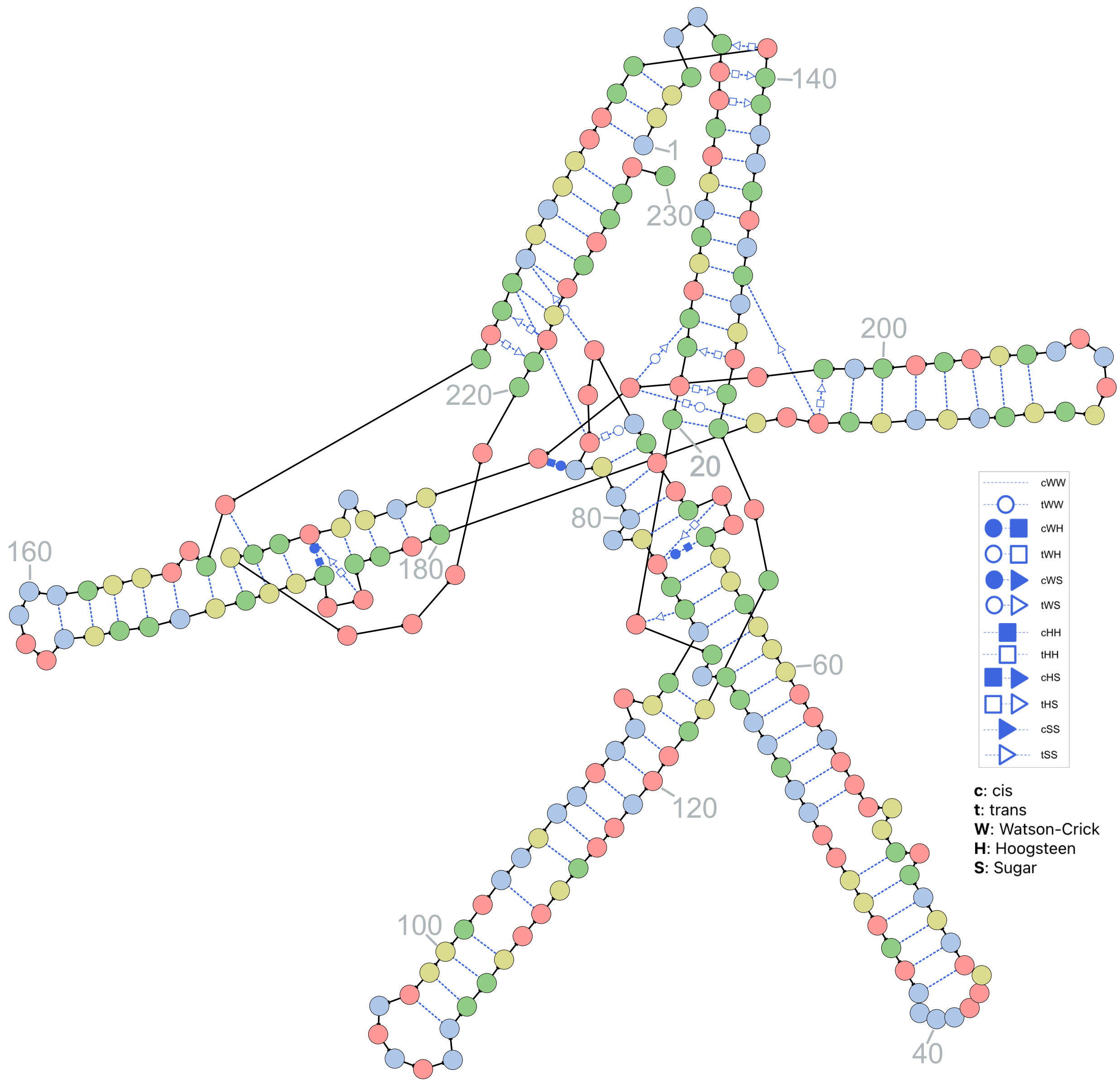

Supplementary Fig. 12: Holo-structure-derived secondary structure and tertiary contacts of the tandem glycine riboswitch. Secondary-structure diagram of the *V. cholerae* tandem glycine riboswitch aptamers annotated using the holo cryo-EM structure. Long-range inter- and intra-aptamer contacts are indicated with Leontis–Westhof symbols[5] where the interaction geometry could be assigned from the model and map. The diagram highlights the major tertiary interactions stabilizing the tandem aptamer architecture, including the reciprocal A-minor-rich contact regions between Apt-I and Apt-II and the conserved inter-aptamer Hoogsteen base pair. Nucleotides are colored according to base, for A (red), G (green), U (blue), and C (yellow) depicted as colored circles. Initial image was made using RNAscape[6] and modified manually.

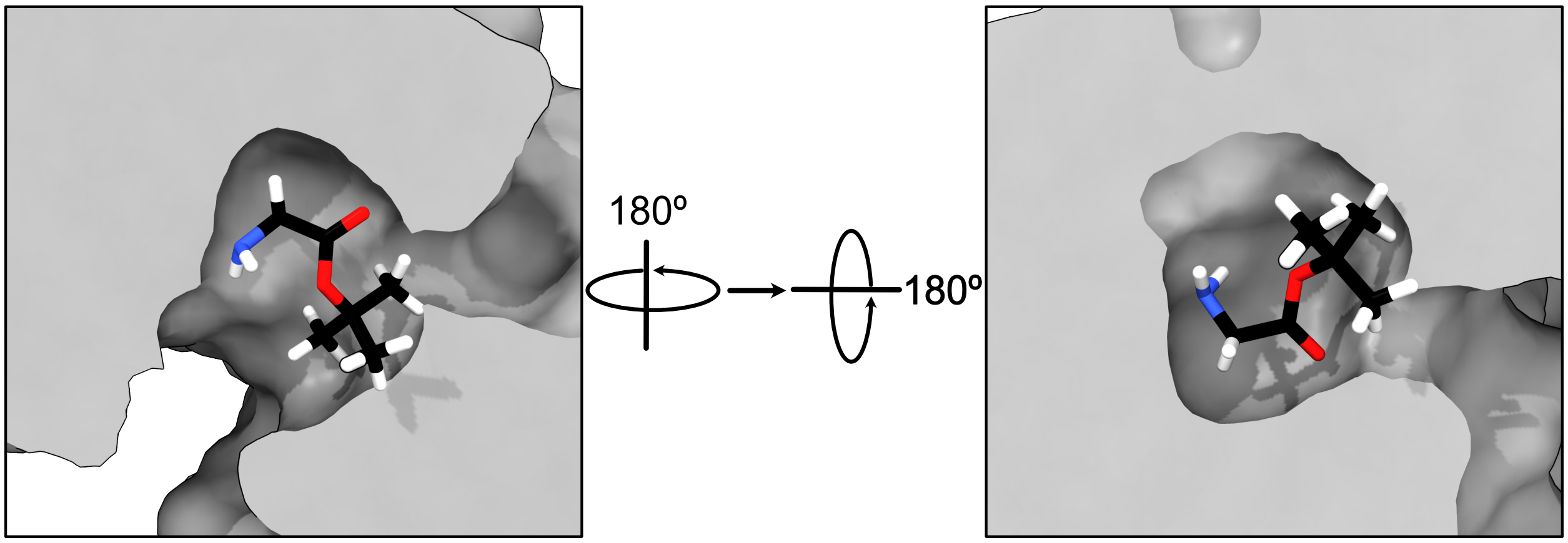

Supplementary Fig. 13: Available space near the glycine carboxyl group in the ligand-binding pocket. Modeled view of the glycine-binding pocket showing that the glycine amino group and Cα position are closely constrained by surrounding RNA, whereas additional space is available near the carboxylate-facing side of the ligand. This arrangement provides a structural rationale for prior biochemical observations that some carboxyl-modified glycine analogs, such as glycine methyl ester and glycine tert-butyl ester, can be accommodated by the glycine riboswitch. The modeled glycine tert-butyl ester position is intended to illustrate the available pocket volume and is not an experimentally determined binding pose.

Supplementary Table 1. Cryo-EM data collection, refinement, and model statistics.

**Cryo-EM data collection and processing**	**holo glyRS – glycine and Mg^{2+}**	**glyRS – Mg^{2+} alone**	**glyRS - no ligand, no Mg^{2+}**
Voltage (kV)	300	300	300
Pixel size (Å)	0.823	0.823	0.823
Electron exposure (e-/Å^2)	59	59	59
Defocus range	-0.6 to -2.4	-0.6 to -2.4	-0.6 to -2.4
Final micrographs used	5,338	5,819	4,312
Particle images (10 models)	384,275	725,637	430,437
Particle images (final)	215,269	207,411	
Resolution (FSC threshold 0.143)	2.86 Å	3.27 Å	
Map sharpening B-factor (Å^2)	-51.8	-68.2	
**Refinement**			
Reference models	PDB: 6WLT, 3P49	Holo glyRS	
Model composition			
Atoms	7477	7422	
Ligands	2	0	
RNA Residues	230	230	
Waters	0	0	
Mg^{2+}	14	12	
CC_{mask}	0.89	0.77	
r.m.s deviations			
Bond lengths (Å)	0.006	0.006	
Bond angles (°)	0.501	0.512	
Clash score	1.75	4.45	
MolProbity score	2.08	2.37	
Pucker outliers	0	0	

Supplementary Movie 1. Stepping motion captured from the 3D Variability Analysis in cryoSPARC. Generated using 20 models at filter resolution of 5 Å.

Supplementary Movie 2. Pivoting motion captured from the 3D Variability Analysis in cryoSPARC. Generated using 20 models at filter resolutions of 5 Å

Supplementary Movie 3. Correlated motion captured by the first principal component (PC1) of the glycine riboswitch. PC1 accounts for 26.5% of the total configurational variance and describes a large-amplitude collective motion of the aptamer domain. The molecular surface is shown interpolated between the two extreme projections along eigenvector 1.

Supplementary Movie 4. Correlated motion captured by the second principal component (PC2) of the glycine riboswitch. PC2 accounts for 15.0% of the total configurational variance and describes a distinct conformational mode orthogonal to PC1. The molecular surface is shown interpolated between the two extreme projections along eigenvector 2.

Supplementary References

1. Torgerson, C. D., Hiller, D. A. & Strobel, S. A. The asymmetry and cooperativity of tandem glycine riboswitch aptamers. *RNA* 26, 564–580 (2020).
2. Goddard, T. D. *et al.* UCSF ChimeraX: Meeting modern challenges in visualization and analysis. *Protein Science* 27, 14–25 (2018).
3. Butler, E. B., Xiong, Y., Wang, J. & Strobel, S. A. Structural Basis of Cooperative Ligand Binding by the Glycine Riboswitch. *Chem. Biol.* 18, 293–298 (2011).
4. Kappel, K. *et al.* Accelerated cryo-EM-guided determination of three-dimensional RNA-only structures. *Nature Methods 2020 17:7* 17, 699–707 (2020).
5. Leontis, N. B. & Westhof, E. Geometric nomenclature and classification of RNA base pairs. *RNA* 7, 499–512 (2001).
6. Mitra, R., Cohen, A. S. & Rohs, R. RNAscape: geometric mapping and customizable visualization of RNA structure. *Nucleic Acids Res.* 52, W354–W361 (2024).
